# An empirical analysis of long-term Brazilian interest rates

**DOI:** 10.1371/journal.pone.0257313

**Published:** 2021-09-10

**Authors:** Tanweer Akram, Syed Al-Helal Uddin

**Affiliations:** 1 Wells Fargo, Washington, DC, United States of America; 2 College of Saint Benedict & Saint John’s University, St. Joseph, MN, United States of America; Universidad de Castilla-La Mancha, SPAIN

## Abstract

This paper empirically models the dynamics of Brazilian government bond (BGB) yields based on monthly macroeconomic data, in the context of the evolution of the key macroeconomic variables in Brazil. The results show that the current short-term interest rate has a decisive influence on the long-term interest rate on BGBs, after controlling for various key macroeconomic variables, such as inflation and industrial production. These findings support John Maynard Keynes’s claim that the central bank’s actions influence the long-term interest rate on government bonds mainly through the current short-term interest rate. These findings have important policy implications for Brazil. This paper relates the findings of the estimated models to ongoing debates in fiscal and monetary policies.

## Introduction

John Maynard Keynes in [1: 352–64] argued that a country’s central bank has a decisive influence on the long-term interest rate on government bonds mainly through its monetary policy. He believed that the central bank’s policy rate sets the current short-term interest rate, which in turn has a crucial effect on the long-term interest rate. This paper examines whether Keynes’s hypothesis that the current short-term interest rate is the key driver of the long-term interest rate holds for Brazil, after controlling for several key macroeconomic variables, such as inflation, and the pace of economic activity or industrial production.

This paper contributes to the literature on the dynamics of government bond yields by examining Brazilian government bond (BGB) yields from a Keynesian perspective. Understanding the empirics of BGB yields is an important question, not just for macroeconomists but also for policymakers and domestic and international investors in Brazilian financial markets. The empirical findings pertaining to the dynamics of BGB yields can be useful for policy purposes. It can be useful for analyzing the effects of fiscal and monetary policy and the monetary transmission mechanism on financial markets. It is also germane for portfolio managers and investors interested in asset allocation in emerging markets and the public sector managers of government debt and Treasury operations. There are only a few empirical analyses of Latin American government bond yields from a Keynesian perspective. Hence, this paper fills a relevant gap in the literature. It provides some valuable insights about the relevance of the Keynesian perspective on government bond yields and financial markets to both macro theorists and policymakers.

The paper is structured as follows. Section II discuss the relevant literature on government bond yields. It also summarizes the literature on the term structure of interest rates. It relates this paper to ongoing debates in the empirical literature on government bond yields. Section III gives a short summary of the evolution of BGB yields with reference to the relevant macroeconomic developments in Brazil. Section IV explains the data and relates the variables to the behavioral equations of the models. It presents the vector error correction method (VECM) applied in the paper. It reports and interprets the empirical findings from the models estimated here. Section V concludes with a discussion of the economic policy implications of the empirical findings for Brazil. S1 Appendix provides the results of unit root tests, cointegration tests, and the optimal lag length section based on several information criteria, while S2 Appendix presents some relevant findings from post estimation statistical tests concerning structural breaks, normality, and autocorrelation, and bidirectional causality.

## Related literature

There is a substantial literature on empirical models of government bond yields [2]. Provides a succinct overview of the debates in the empirical literature on government bond yields. There are two contending viewpoints in the literature.

The dominant view is that a higher government debt or deficit ratio leads to higher government bond yields. This viewpoint is represented by the neoclassical perspective, such as [3–15]. This view is based on the loanable funds theory. According to this theory, the interest rate is simply the price of loanable funds. It holds that the supply of loanable funds (or saving) is discouraged (encouraged) by low (high) interest rate. Increased government net borrowing leads to higher demand for funds. Given a supply schedule, higher demand for funds raises the equilibrium interest rate.

There are three dominant theories of the term structure of interest rates, namely (1) the pure expectation theory, (2) the market segmentation theory, and (3) the liquidity premium theory. The pure expectations theory maintains investors in bonds are generally indifferent to the maturity tenor of bonds and that bonds are perfect substitutes for one another. In the pure expectation theory of the term structure, the forward rate represents the expectation for future spot rate [16]. Is a modern representation of the pure expectations theory, whereas [17] is an early proponent of the same. Under the market segmentation theory, bonds of different maturity tenors are not substituting for one another. This theory holds that the interest rate on bonds of each maturity tenors is determined by the demand and the supply of bonds in the relevant segment and hence that there is no necessary connection between the long-term interest rate and the short-term interest rate. The market segment theory is articulated in [18]. The liquidity premium theory maintains that bonds of different maturity tenors are imperfect substitutes for one another. Hence, the long-term interest rate does not just depend on the spot rate and the expected spot rates in the future, but also reflects some term premium required to compensate the investor because of the uncertainty concerning the future path of the short-term interest rate and other factors [19]. Proposed the liquidity premium theory of the term structure of interest rates.

Although the main theories of the term structure differ from one another, these theories are derived from the loanable funds theory. However, the scope of loanable funds market varies among the theories. There is a single loanable funds market for all types of bonds in the pure expectation theory. However, in the market segmentation theory, there are separate loanable funds markets for particular segments of the bond market. In liquidity premium theory there is an integrated loanable funds market, but bonds of different maturities are imperfect substitutes and hence the long-term interest rate does not merely reflect the market expectation of future spot rates but commands a term premium reflecting uncertainty about the future. There is a substantial empirical literature on the term structure of interest rates, such as [20–24]. A more detailed overview of the theory and the empirics of the term structure of interest rates are provided in [25–26].

In contrast to the dominant views of the term structure of interest rates, there is a minority view which maintains that the central bank’s action, particularly its policy rate, is the key driver of government bond yields. This viewpoint originates from Keynes [1, 27], who was inspired by Riefler’s empirical analysis [28] of the long-term interest rate in the United States in the 1920s and his own observation about the behavior of the long-term interests in the United Kingdom in the same time period. Keynes [27: 167] firmly rejects the view that the interest rate is “a return to saving or waiting” or “the ‘price’ which brings into equilibrium the demand for resources to investment with the readiness to abstain from present consumption.” Instead, Keynes [27] maintains that the interest rate is “the reward for parting with liquidity for a specified period” which “equilibrates the desire to hold wealth in the form of cash with the available quantity of cash.” Keynes [1: 302–3] maintained that “the long-term rate will respond to the wishes of a Currency Authority which will be exerting its direct influence … mainly on the short-term rate.” Whereas expectations theory holds that the long-term interest depends on the expected path of the short-term interest rate, for Keynes “the influence of the [current] short-term rate of interest on the long-term rate is much greater than anyone … would have expected.”

The Keynesian approach to interest rate is represented in [1, 29–45]. The Keynesian approach on the dynamics of government bond market draws on a wide range of theoretical arguments in the literature, such as [46–53]. It also draws on various empirical analysis and policy discussions, such as [54–59].

This paper contributes to the literature in several propitious ways. First, it econometrically models government bond yields in Brazil, a major emerging market country. It is useful to examine whether Keynes’s conjecture holds for an emerging market country, such as Brazil. Second, it extends the research program of the Keynesian approach on modelling government bond yields to the case of Brazil. Third, it relates the developments in the BGB market to macroeconomic fundamentals and recent economic developments in Brazil. Fourth, it discerns the implications of the findings from the empirical modelling of the dynamics of the government bond market in Brazil for fiscal and monetary policies not only in Brazil but also for emerging market countries that issues government debt in their own currencies and exercises monetary sovereignty. This paper contributes to the ongoing debates on the empirical analysis of dynamics of government bond yields in the growing literature on government bond markets in emerging market countries. See [30–31, 60–62] for examples of the current debates in the literature on government bond yields in emerging markets.

## Government bond yields and macroeconomic developments in Brazil

The figures below show the evolution of the relevant macroeconomic variables related to government bond yields in Brazil from 2007 to 2018. The shaded areas in light grey in the figures are the periods of recession. Since 2014 the Brazilian economy has slowed down noticeably. In recent years, Brazil has suffered from political uncertainty, weakness in growth, elevated inflation, and a deprecation of the currency and volatility in the exchange rate for the currency [63–67], even though Brazil is a country with tremendous potential [68].

**Fig 1:**
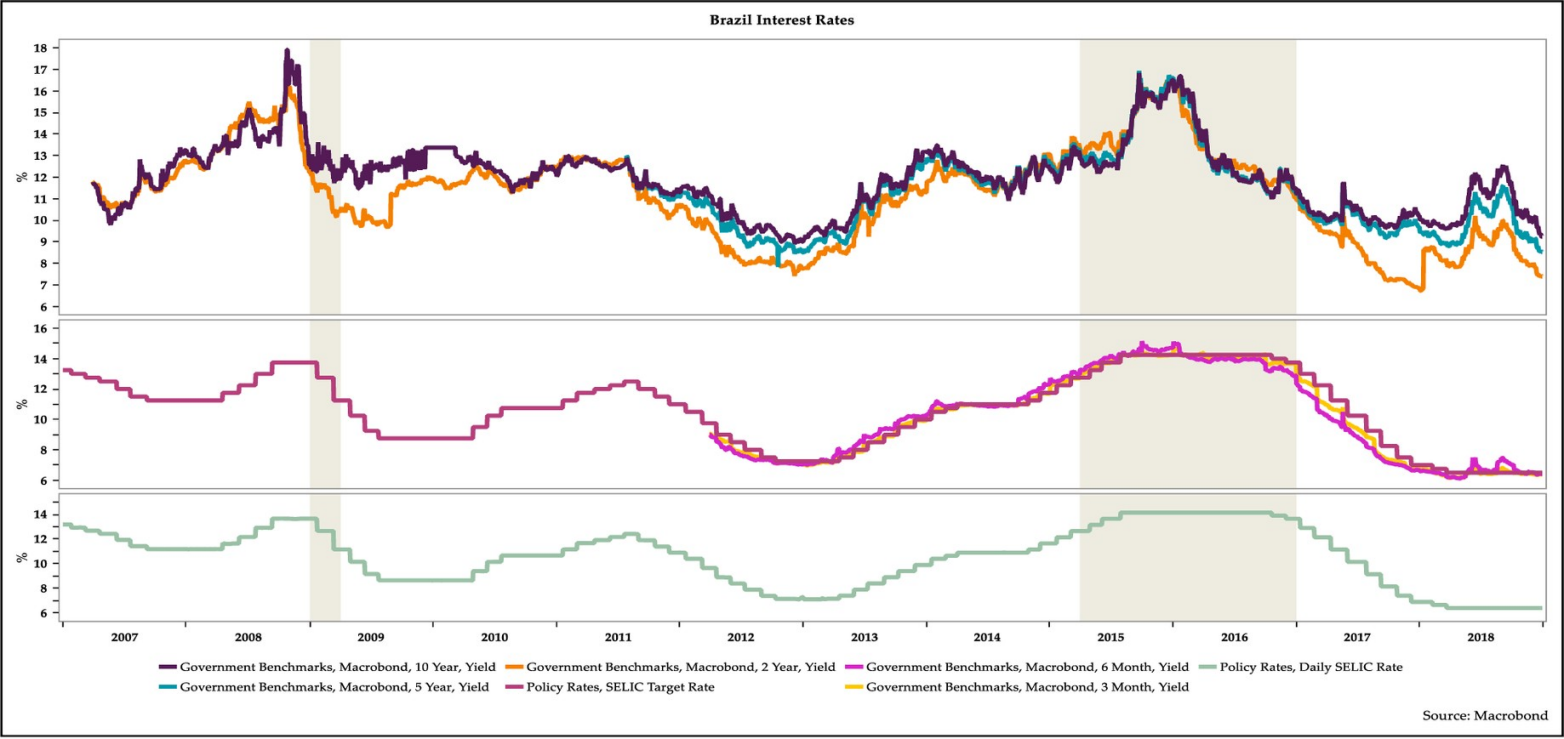
The evolution of key interest rates in Brazil, 2007–2018.

Fig 1 shows the evolution of key interest rates in Brazil. Long-term interest rates on government bonds rose sharply from around 11 percent in early 2007 to almost 18 percent by mid-2008 but fell noticeably just before the onset of the recession in 2009 as the Banco Central do Brasil (BCB), the country’s central bank, cut its policy rate. Long-term interest rates were fairly steady from 2009 to mid-2011, even as the BCB started hiking the policy rate in mid-2010. Long-term interest rates began to decline from mid-2011 to early 2013 as the BCB gradually reduced its policy rate. As the BCB renewed tightening monetary policy, long-term interest rates rose from mid-2013 to mid-2015. Long-term interest rates started declining in anticipation of a reduction in the BCB’s policy rate in late 2015. This decline generally continued as the BCB lowered its policy rate from late 2016 to early 2018. However, long-term interest rates initially rose in mid-2018 even though the BCB held the policy rate steady. Eventually by late 2018 long-term interest rates began to decline.

**Fig 2:**
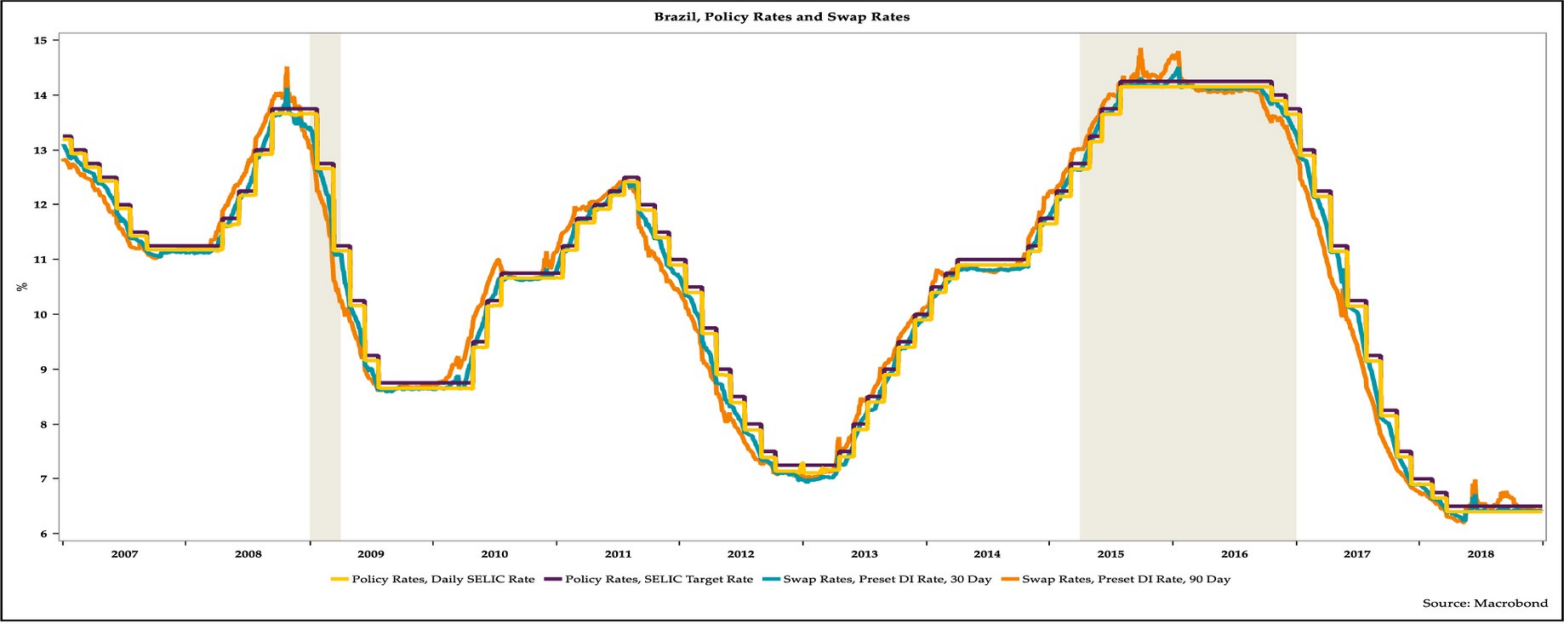
The evolution of policy rates and swap rates in Brazil, 2007–2018.

Fig 2 displays the evolution of targeted and effective policy rates and short-term swap rates. It reveals that short-term swap rates are tightly connected with the BCB’s policy rate.

**Fig 3:**
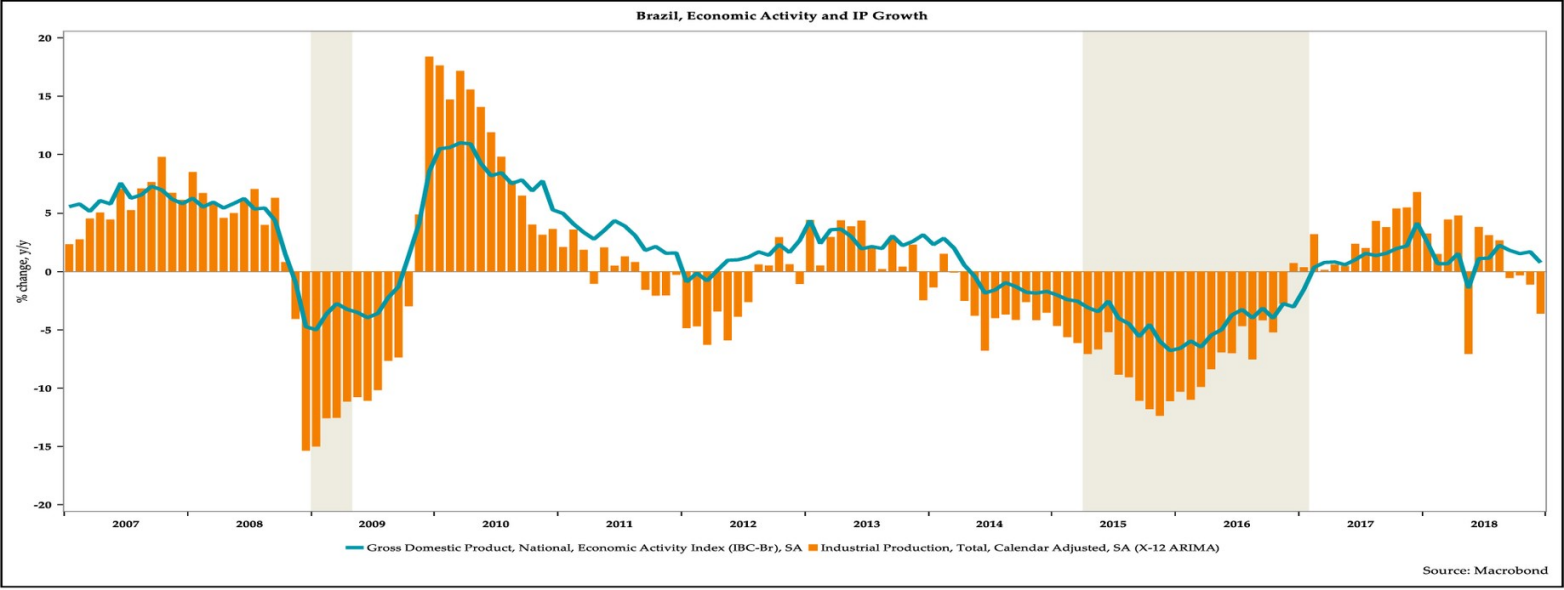
The evolution of economic activity and industrial production in Brazil, 2007–2018.

Fig 3 shows the evolution of the economic activity, as a measured by year-over-year changes in monthly real GDP and monthly industrial production.

**Fig 4:**
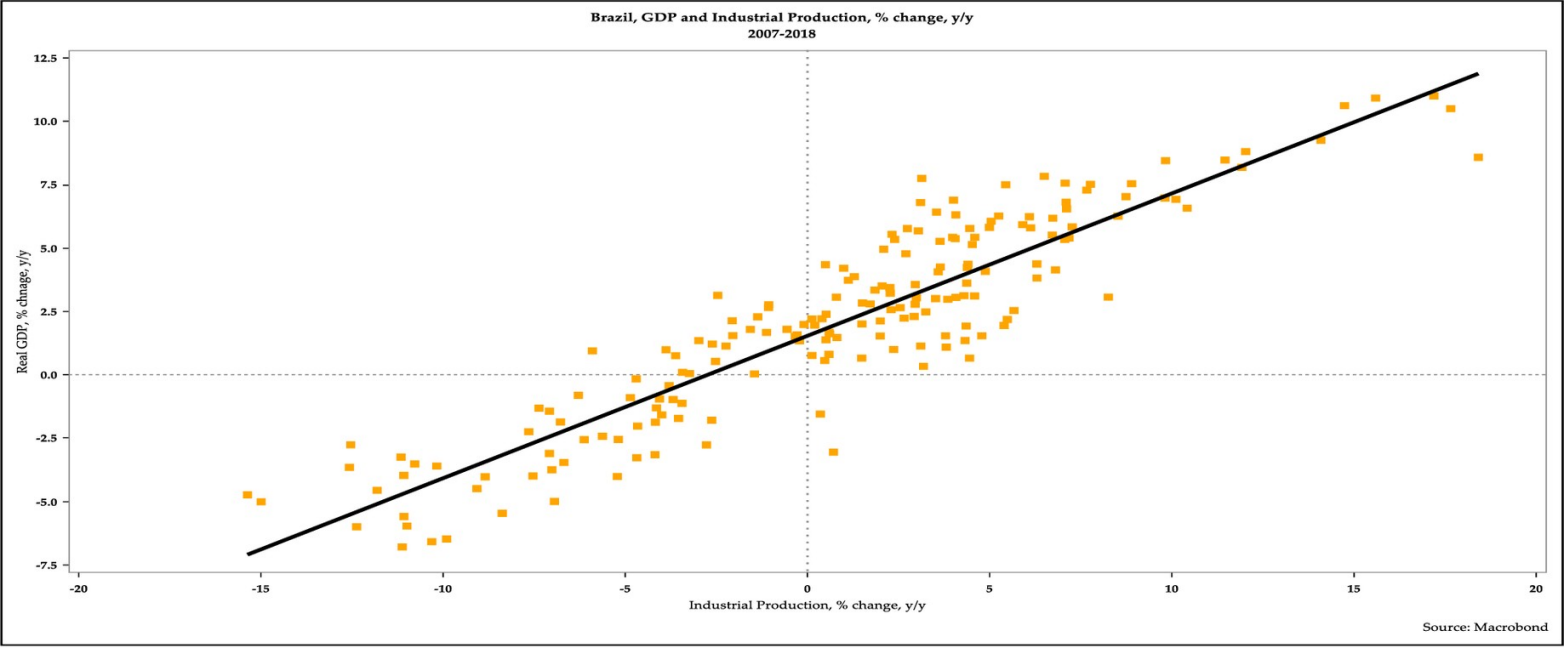
Scatterplot of real GDP growth and industrial production growth in Brazil, 2007–2018.

The scatterplot in Fig 4 affirms that the year-over-year changes in monthly real GDP and industrial production are strongly correlated.

**Fig 5:**
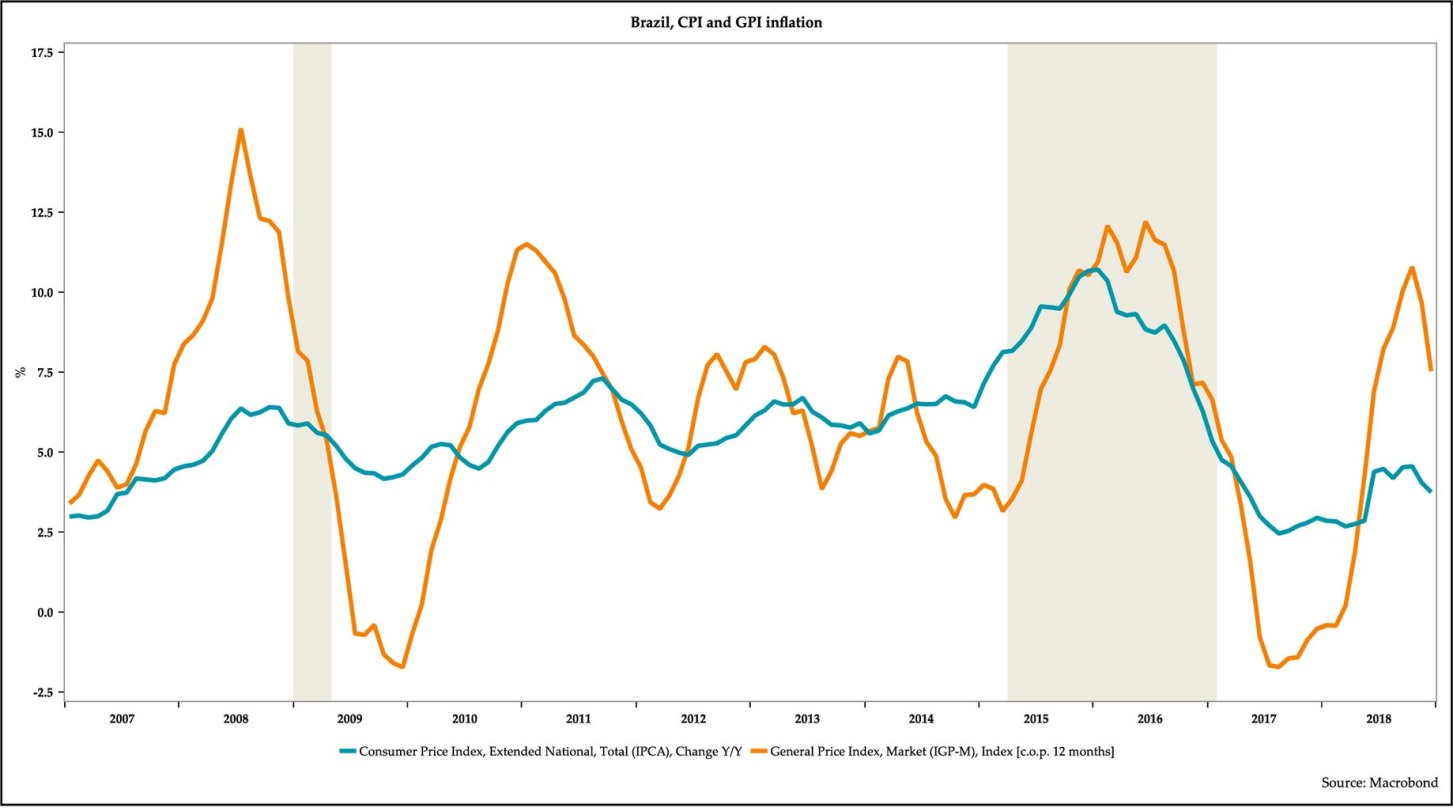
The evolution of inflation in Brazil, 2007–2018.

Fig 5 depicts inflation in Brazil as measured by two different indicators of inflation. These indicators are the year over year changes in the consumer price index (CPI) and the general price index (GPI). The GPI is more volatile than the CPI. CPI inflation rose steadily from 2.5 percent in 2007 to over 10.0 percent by 2016. However, CPI inflation has declined in recent years and was hovering around 3.0 percent by late 2018. GPI inflation has been quite volatile.

**Fig 6:**
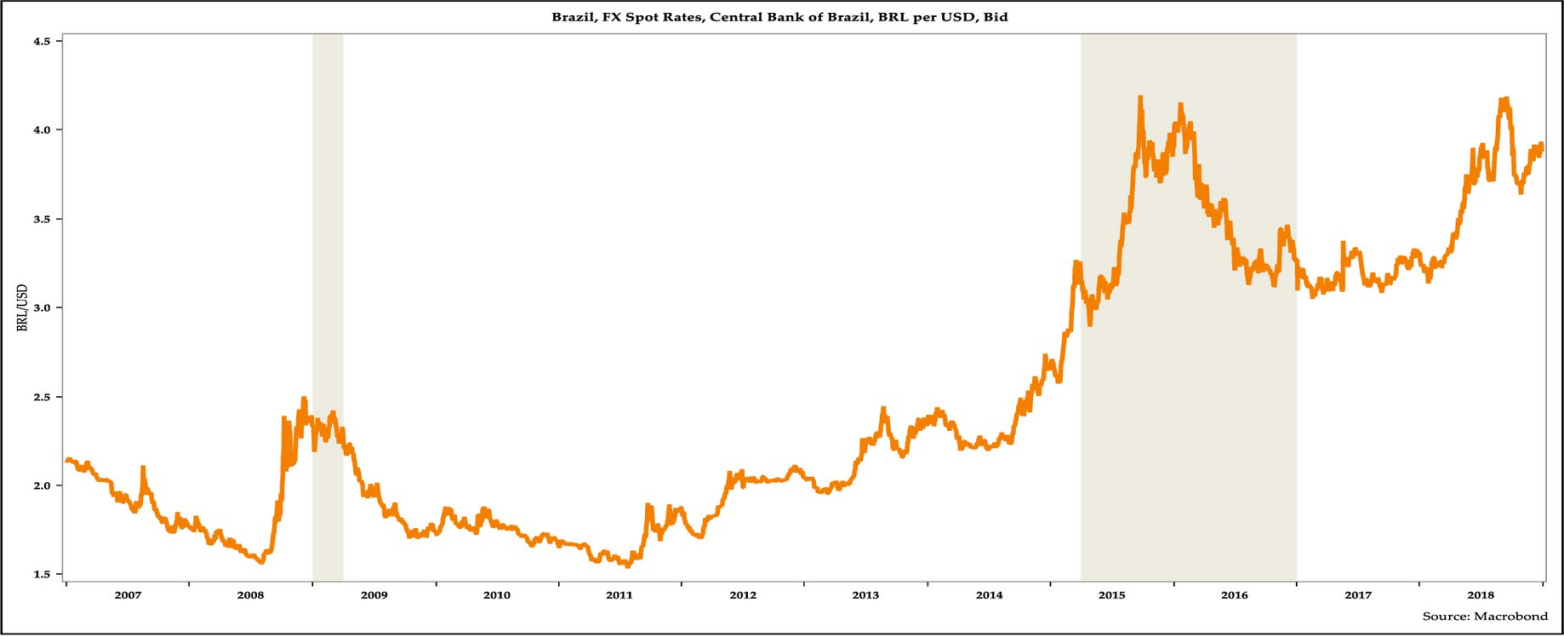
The evolution of the Brazilian real, 2007–2018.

Fig 6 shows the evolution of the Brazilian real. The real depreciated with the global financial crisis. It deprecated ahead of the Brazilian recession in early 2009, but it appreciated from mid-2009 to till 2011 as the economy recovered. However, since late 2011 the Brazilian real depreciated steadily till early 2016. It appreciated in early 2016. It was stable for more than a year. However, in 2018, it again depreciated.

**Fig 7:**
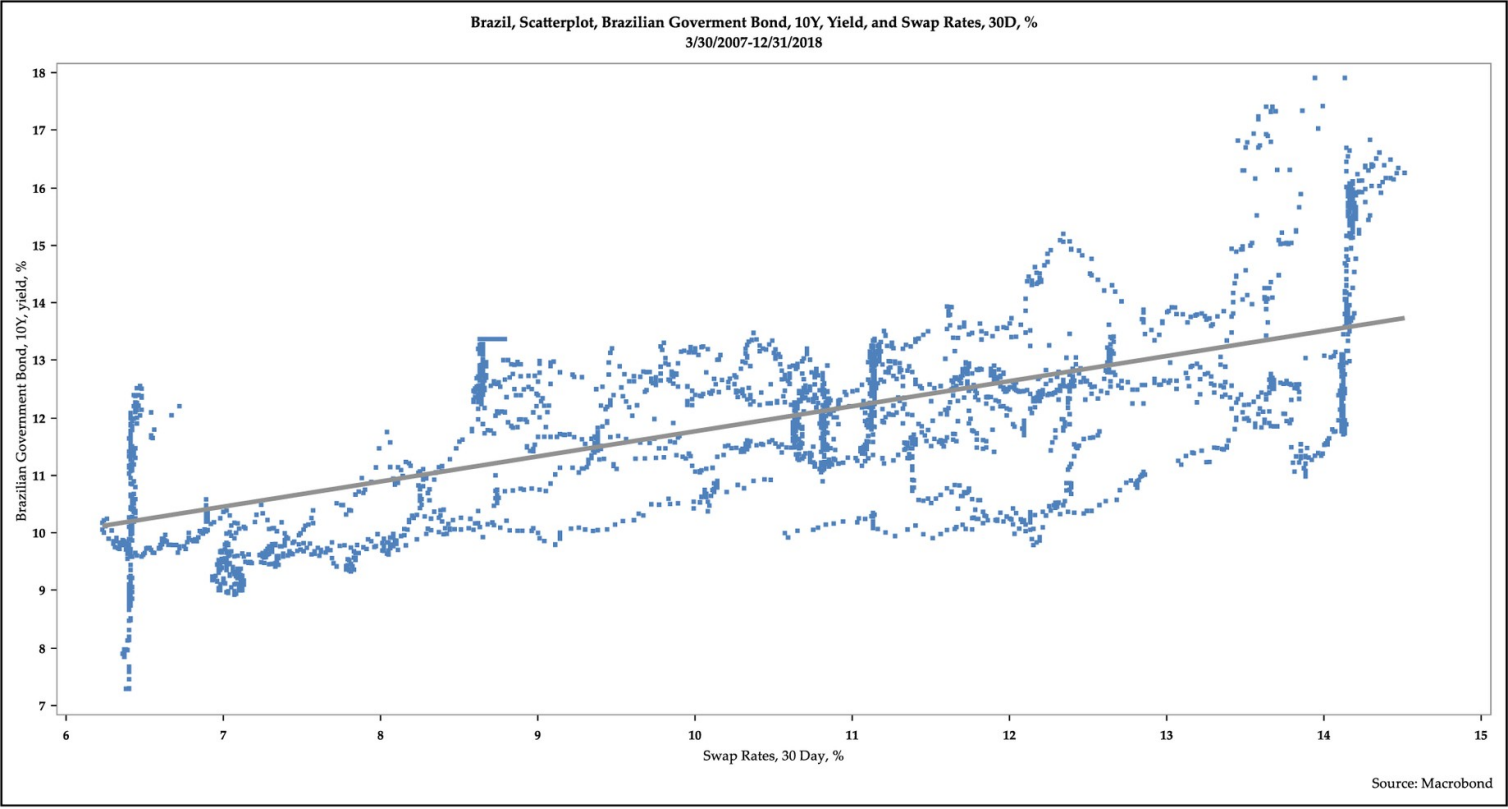
Scatterplot of the yields of 10-year Brazilian government bonds and 30-day swaps.

**Fig 8:**
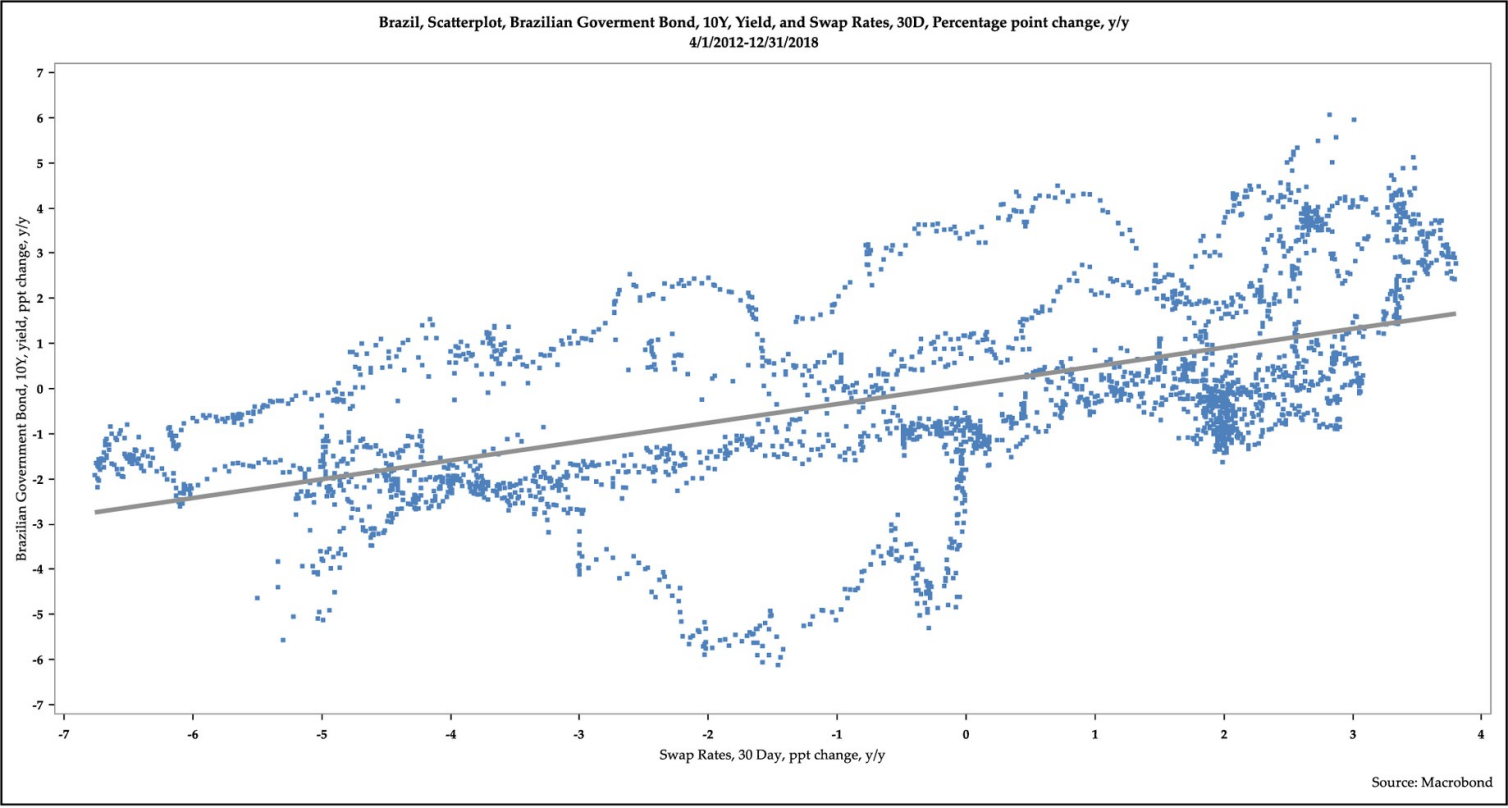
Scatterplot of the year-over-year percentage point changes in the yields of 10-year Brazilian government bonds and 30-day swaps.

Fig 7 is a scatterplot of the yields of 10-year BGB and 30-day swap. Fig 8 is a scatterplot of the year-over-year percentage point changes in yields of 10-year BGB and 30-day swap. Additional scatterplots are available in the working paper.

These scatterplots demonstrate some fascinating patterns. First, the yields of long-term BGBs and short-term securities, as measured by swap rates, are positively and tightly correlated. Second, the changes of the yields of long-term BGBs and short-term interest rates, as measured by swap rates, are also positively correlated, though less so than in the levels of the yields. Third, these correlations weaken somewhat as the maturity tenor of the long-term BGBs rise.

## Data, methodology, model, and empirical results

### Data

Table 1 provides a summary of the data and the variables used in this paper. The first column gives the variable labels. The second column provides the data description and date ranges of the data. The third column shows the original frequency of the data and indicates whether the original data have been converted to monthly frequency. The final column lists the sources of the data.

**Table 1:** Summary of the data and the variables.

Variable labels	Data description, date ranges	Frequency	Sources
***Policy rates***			
**SELIC**	SELIC daily rate, effective, %; 1/1/2007 to 12/1/2018	Daily; converted to monthly	Central Bank of Brazil; [69]
***Short-term interest rates***			
**SWAP30D**	Swap rate, preset rate, 30 day, %; 1/1/2007 to 12/1/2018	Daily; converted to monthly	Central Bank of Brazil; [69]
***Government bond yields***			
**GB2Y**	Government bonds, 2-year, yield, %; 4/1/2007 to 12/1/2018	Daily; converted to monthly	Brazilian Financial and Capital Markets Association; [69]
**GB10Y**	Government bonds, 10-year, yield, %; 4/1/2007 to 12/1/2018	Daily; converted to monthly	Brazilian Financial and Capital Markets Association; [69]
***Inflation***			
**CPI**	Consumer price index, extended national, total, % change, y/y; 1/1/2007 to 12/1/2018	Monthly	Brazilian Institute of Geography and Statistics; [69]
**GPI**	General price index, market, index, % change, y/y; 1/1/2007 to 12/1/2018	Monthly	Getulio Vargas Foundation (Brazilian Institute of Economy); [69]
***Pace of economic activity***			
**GDP**	Real gross domestic product, national, economic activity, index, SA, % change, y/y; 1/1/2007 to 12/1/2018	Monthly	Central Bank of Brazil; [69]
**IP**	Industrial production, total, calendar adjusted, index, seasonally adjusted, % change, y/y; 1/1/2007 to 12/1/2018	Monthly	Brazilian Institute of Geography and Statistics; [69]

The variable for the BCB policy rates is the Sistema Especial de Liquidação e de Custódia (SELIC) daily effective rate. The variable for the short-term interest rate is the 30-day swap rate. The variables for long-term interest rates are the yields of 2-year and 10-year BGBs. The variables for inflation rates are year-over-year percentage changes in the CPI and the GPI. The variables for economic activity are year over year changes in the measures of real gross domestic product (GDP) and the index of industrial production.

Monthly time series data are used in this paper. Some daily time series data have been converted to monthly series. The time-series data for most of the variables are from January 2007 to December 2018, which amounts to 12 years of monthly data, consisting of more than 140 monthly observations.

### Methodology and empirical results

In this paper, the primary goals are to (1) test Keynes’s hypothesis concerning the relationship between the current short-term interest rate and the long-term interest rate, and (2) understand the short-run and the long-run relations among BGB yields, the short-term interest rate, and other variables. The vector error correction model (VECM) is used to test the hypothesis and estimate coefficients. The specification of the behavioral equations presented here are consistent with Keynesian models of government bonds yields, such as [29–39]. These behavioral equations are convenient and readily render themselves to empirical modeling.

Several tasks are undertaken to validate the estimation process before undertaking estimation with the VECM. First, tests are conducted for unit roots, followed by check for cointegration among the variables. Second, the optimal lag-length for the equations is determined using an appropriate statistical technique. Third, the VECM model is applied based on the Johansen cointegration [70] with optimal lag length.

The results of unit root tests for the variables and their cointegrating behavior based on lag length selection are provided in S1 Appendix. The results of various statistical post estimation tests, such as structural break, normality, and autocorrelations, are discussed in S2 Appendix.

### Vector error correction model (VECM)

VECMs are appropriate when variables are first difference stationary while nonstationary in their levels. The unit roots tests, provided in S1 Appendix, show that these series are non-stationary in their levels but are stationary in their first differences. Hence, these variables are integrated of order I(1). VECMs can be used to estimate the short-term and the long-term relationships among such variables. Moreover, the adjustment factors from short-term to long-term dynamics can also be estimated.

The next three tables explain the results of VECM model. The analysis is based on the long-run relationship, the short-run relationship, and the adjustment from short-run deviation to long-run equilibrium.

Table 2 shows the estimation results from VECM model. It reveals the long-term relationship among the variables. Table 2 presents the long-term relationship based on Eqs 1 and 2.

**Table 2:** Long-run relationship among variables for Eq 1 and Eq 2.

Variables	Eq 1	Eq 2
GB2Y	1	1
SWAP30D	-0.511*** (0.099)	-0.868*** (0.197)
GPI	0.195*** (0.059)	0.624*** (0.131)
GDP	0.249*** (0.064)	
Trend	0.028*** (0.006)	0.024** (0.009)

Notes: Standard errors in parentheses.

*** p<0.01

** p<0.05

* p<0.1.


$$\Delta Z_{t}=\alpha \beta^{\prime }Z_{t-1}+\overset{p-1}{\underset{i=1}{\sum }}\Gamma_{i}\Delta Z_{t-i}+v+\delta t+\epsilon_{t}$$ (1)

Where *Z* = [*GB*2*Y*, *SWAP*30*D*, *GPI*, *GDP*]′

$$\Delta Z_{t}=\alpha \beta^{\prime }Z_{t-1}+\sum_{i=1}^{p-1}\Gamma_{i}\Delta Z_{t-i}+v+\delta t+\epsilon_{t}$$ (2)

Where *Z* = [*GB*2*Y*, *SWAP*30*D*, *GPI*]′

From the cointegrating rank test (provided in Table A4 in S1 Appendix, part A), Eq 1 has a rank of 1, which implies one error correction equation. It is evident that all these variables are statistically significant, which says that GB2Y has a long-term causality with SWAP30D, GPI, and GDP. The result indicates that there is a positive relationship between swap rate (30 days) and government bonds yields (2-year yield) in the long term. This implies that if the SWAP30D rate increases, then government bond yields for two-year maturity rate also increases. There is a negative association between real GDP and bond yields and between GPI and bond yields. This indicates that if real GDP or GPI increases, then government bond yields decrease. In column 2 reports the result excluding GDP from the regression. It is evident from the table that even if GDP is excluded from the analysis, then all the variables are statistically significant, and that model fits well. Interestingly, after dropping the variable GDP, the SWAP30D rate has a larger coefficient compared to the previous model.

In Table 3, the dependent variable is GB10. Column 2 shows the result based on Eq 3, while column 3 shows the result based on Eq 4. The swap rate is statistically insignificant, though it has the expected sign. Here GDP, GPI, and the linear trend variables are statistically significant. In column 4, even after dropping GDP from the regression, the swap rate is still statistically insignificant.

**Table 3:** Long-run relationship among variables for Eq 3 and Eq 4.

Variables	Eq 3	Eq 4
GB10Y	1	1
SWAP30D	-0.045 (0.178)	-0.369 (0.233)
GPI	0.357*** (0.106)	0.586*** (0.150)
GDP	0.425*** (0.115)	
Trend	0.040*** (0.006)	0.024** (0.011)

Notes: Standard errors in parentheses.

*** p<0.01

** p<0.05

* p<0.1.


$$\Delta Z_{t}=\alpha \beta^{\prime }Z_{t-1}+\sum_{i=1}^{p-1}\Gamma_{i}\Delta Z_{t-i}+v+\delta t+\epsilon_{t}$$ (3)

Where *Z* = [*GB*10*Y*, *SWAP*30*D*, *GPI*, *GDP*]′

$$\Delta Z_{t}=\alpha \beta^{\prime }Z_{t-1}+\overset{p-1}{\underset{i=1}{\sum }}\Gamma_{i}\Delta Z_{t-i}+v+\delta t+\epsilon_{t}$$ (4)

Where *Z* = [*GB*10*Y*, *SWAP*30*D*, *GPI]′*

Table 4 presents the speed of adjustments to the long-term equilibrium from the short-term deviation. It is evident that GB2Y is statistically significant with the expected (negative) sign in columns 2 and 5 and row 2 (with error correction). This implies that short-term deviation from the long-term equilibrium is adjusted by 0.191 and 0.23 percentage points each month, respectively. It is also evident that SWAP30D and GPI are not statistically significant, but GDP is statistically significant with the expected sign. Thus, the GDP growth rate has a positive effect on long-term convergence. To find the short-term causality among variables, it is useful to look at the lag coefficients for each variable. In Table 4, for the GB2Y (columns 2 and 5), none of the lags of GB2Y are statistically significant, but both lags of SWAP30D are statistically significant. Apart from that, the first lag of both the GPI and GDP variables is statistically insignificant, whereas the second lag is statistically significant. Therefore, SWAP30D, GPI, and GDP have short-run causality with GB2Y after the first lag. It implies that an increase in the previous months’ GB2Y yield does not influence the current month’s GB2Y increase. However, an increase in the last month’s SWAP30D is associated with an increase in the current month’s GB2Y yield. Similarly, GDP and GPI increase with a two-month lag increases the current month’s GB2Y yield.

**Table 4:** Speed of adjustment and short-run relationship from VECM for Eq 1 and Eq 2.

	Eq 2			Eq 1			
VARIABLES	ΔGB2Y	ΔSWAP30D	ΔGPI	ΔGB2Y	ΔSWAP30D	ΔGPI	ΔGDP
	Speed of adjustment						
Error Correction	-0.191***	-0.000462	-0.153***	-0.226***	0.00753	-0.0951	-0.244***
	(0.0403)	(0.0135)	(0.0519)	(0.0479)	(0.0162)	(0.0644)	(0.0904)
	Short-run relationship						
ΔGB2Y(t-1)	0.120	0.102***	0.285**	0.128	0.0941***	0.275**	-0.0158
	(0.0860)	(0.0287)	(0.111)	(0.0857)	(0.0289)	(0.115)	(0.162)
ΔGB2Y(t-2)	-0.0197	0.0375	-0.0576	-0.0103	0.0369	-0.0595	0.0341
	(0.0889)	(0.0297)	(0.114)	(0.0871)	(0.0294)	(0.117)	(0.165)
ΔSWAP30D(t-1)	0.452*	0.376***	0.248	0.511**	0.366***	0.252	0.191
	(0.254)	(0.0849)	(0.327)	(0.253)	(0.0855)	(0.341)	(0.478)
ΔSWAP30D(t-2)	0.424*	0.332***	0.449	0.645**	0.345***	0.377	0.248
	(0.245)	(0.0821)	(0.316)	(0.252)	(0.0852)	(0.339)	(0.477)
ΔGPI(t-1)	-0.0140	0.0186	0.706***	-0.00699	0.0110	0.708***	0.131
	(0.0678)	(0.0227)	(0.0873)	(0.0674)	(0.0228)	(0.0907)	(0.127)
ΔGPI(t-2)	0.140**	0.0248	-0.0567	0.165**	0.0237	-0.0770	-0.0926
	(0.0704)	(0.0235)	(0.0907)	(0.0701)	(0.0237)	(0.0943)	(0.132)
ΔGDP(t-1)				0.0651	0.0237	0.0928	0.119
				(0.0438)	(0.0148)	(0.0588)	(0.0827)
ΔGDP(t-2)				0.102**	0.0182	0.000118	0.229***
				(0.0445)	(0.0150)	(0.0599)	(0.0841)
Constant	-0.0139	-0.00785	0.0174	0.0173	-0.00589	0.0328	-0.0290
	(0.0501)	(0.0167)	(0.0645)	(0.0493)	(0.0167)	(0.0663)	(0.0932)
Observations	138	138	138	138	138	138	138
P>chi2	0.0001	0.0000	0.0000	0.0000	0.0000	0.0000	0.0017
R-square	0.1953	0.6945	0.5097	0.2406	0.706	0.4958	0.1814

Notes: Standard errors in parentheses.

*** p<0.01

** p<0.05

* p<0.1.

When considering long-term government bond yields, GPI is statistically significant, meaning that longer-term inflation is an important factor in determining government bond yields. For example, columns 2 and 5 of Table 5, show that the yields of GB10Y adjust respectively by a factor of .101 and .14 percentage points each month to attain long-run equilibrium.

**Table 5:** Speed of adjustment and short-run relationship from VECM for Eq 3 and Eq 4.

	Eq 4			Eq 3			
VARIABLES	ΔGB10Y	ΔSWAP30D	ΔGPI	ΔGB10Y	ΔSWAP30D	ΔGPI	ΔGDP
	Speed of adjustment						
Error Correction	-0.101***	-0.0112	-0.0910***	-0.137***	-0.0109	-0.0716*	-0.165***
	(0.0265)	(0.00728)	(0.0264)	(0.0357)	(0.00991)	(0.0373)	(0.0533)
	Short-run relationship						
ΔGB10Y(t-1)	0.00185	0.0560**	0.242***	0.0351	0.0566**	0.248***	-0.0714
	(0.0837)	(0.0230)	(0.0833)	(0.0831)	(0.0231)	(0.0868)	(0.124)
ΔGB10Y(t-2)	-0.156*	0.00696	-0.184**	-0.146*	0.0101	-0.181**	-0.109
	(0.0868)	(0.0239)	(0.0864)	(0.0857)	(0.0238)	(0.0896)	(0.128)
ΔSWAP30D(t-1)	0.284	0.458***	0.328	0.272	0.455***	0.387	0.0459
	(0.301)	(0.0826)	(0.299)	(0.297)	(0.0825)	(0.311)	(0.443)
ΔSWAP30D(t-2)	0.419	0.339***	0.260	0.640**	0.372***	0.229	0.154
	(0.300)	(0.0823)	(0.298)	(0.305)	(0.0847)	(0.319)	(0.456)
ΔGPI(t-1)	-0.0297	0.0272	0.723***	-0.0250	0.0220	0.736***	0.155
	(0.0850)	(0.0234)	(0.0846)	(0.0844)	(0.0234)	(0.0882)	(0.126)
ΔGPI(t-2)	0.0710	0.0296	-0.0608	0.101	0.0321	-0.0765	-0.106
	(0.0881)	(0.0242)	(0.0877)	(0.0881)	(0.0244)	(0.0921)	(0.131)
ΔGDP(t-1)				-0.0165	0.0228	0.0746	0.104
				(0.0548)	(0.0152)	(0.0573)	(0.0818)
ΔGDP(t-2)				0.143***	0.0227	0.00883	0.213***
				(0.0552)	(0.0153)	(0.0577)	(0.0824)
Constant	-0.00478	-0.00977	0.00651	0.0268	-0.00471	0.0286	-0.0344
	(0.0624)	(0.0171)	(0.0621)	(0.0613)	(0.0170)	(0.0641)	(0.0915)
Observations	138	138	138	138	138	138	138
P>chi2	0.0042	0.0000	0.0000	0.0007	0.0000	0.0000	0.0003
R-square	0.1481	0.6809	0.5473	0.1931	0.1931	0.5273	0.2073

Notes: Standard errors in parentheses.

*** p<0.01

** p<0.05

* p<0.1.

Table 5 shows the short-run relationship among variables based on Eq 3 and Eq 4. From column 2, the second lag of GB10Y is statistically significant. This implies that the current month’s GB10Y is negatively affected by the 2-months lagged GB10Y yield at 0.15 percentage points. The second lags of SWAP30D and GDP are statistically significant and positively influence the current month’s GB10Y yield.

This empirical exercise in this paper shows a positive relationship between the government bond yield and the short-term interest rate measured by SWAP30D. The association is statistically significant in the front end of the yield curve but not so in the back end of the yield curve, though signs are always positive. The sign in the short-run to long-run deviation is negative, as expected. The findings provide some qualified support for Keynes’s contention.

### An alternative specification

An alternative specification is also executed for determining the long-term interest rate with a different set of independent variables: SELIC rate, CPI, and IP. The unit-roots test, lag-lengths, and cointegration results are provided in the working paper. The following tables describe the short-run and long-run relationship using these variables.


$$\Delta Z_{t}=\alpha \beta^{\prime }Z_{t-1}+\sum_{i=1}^{p-1}\Gamma_{i}\Delta Z_{t-i}+v+\delta t+\epsilon_{t}$$

Where

Z=[GB2Y,SELIC,CPI,IP]′ (5)

Z=[GB2Y,SELIC,CPI]′ (6)

Z=[GB10Y,SELIC,CPI,IP]′ (7)

Z=[GB10Y,SELIC,CPI]′ (8)

Table 6 presents the long-run relationship among these variables. The rank test confirms that there is only one cointegrating relation. Each column represents a long-run relationship among the variables. For example, in column 2 (based on Eq 5), all the variables are statistically significant. The GB2Y has a long-term relationship with SELIC, CPI, and IP. For example, for a 1 percent increase in SELIC rate, GB2Y increases by 0.90 percentage points in the long run. With GB10Y (based on Eq 7) as a dependent variable and the same independent variables, all the variables are statistically significant except for the trend term. It implies that a 1 percent increase in the SELIC rate increases the GB10Y by 0.70 percentage points in the long run.

**Table 6:** Long-run relationship under alternative specification.

Variables	Eq [5]	Eq [7]
GB2Y	1	---
GB10Y	---	1
SELIC	-0.893*** (0.119)	-0.705*** (0.185)
CPI	0.748*** (0.207)	1.237*** (0.322)
IP	0.183*** (0.045)	0.299*** (0.070)
Trend	0.011** (0.005)	0.012 (0.008)

Notes: Standard errors in parentheses.

*** p<0.01

** p<0.05

* p<0.1.

Table 7 shows the speed of adjustment coefficients and short-run relationship among variables following Eq 5. For example, column 2 reports the estimation with GB2Y as a dependent variable, where long-run equilibrium from a short-run disequilibrium is achieved at a rate of 0.137 percentage points each month for GB2Y. Similarly, for GB10Y (based on Eq 7), the convergence speed from a short-run disequilibrium to a long-run equilibrium is 0.138 percentage points, as shown in column 2 in Table 8.

**Table 7:** Speed of adjustment and short-run relationship from VECM for Eq 5.

VARIABLES	ΔGB2Y	ΔSELIC	ΔCPI	ΔIP
	Speed of adjustment			
Error Correction	-0.137**	0.0846***	-0.0395	-0.431
	(0.0550)	(0.0245)	(0.0266)	(0.273)
	Short-run relationships			
ΔGB2Y(t-1)	0.152	0.0901**	0.115**	0.757
	(0.0930)	(0.0415)	(0.0450)	(0.462)
ΔGB2Y(t-2)	-0.0114	0.0254	0.0192	0.182
	(0.0948)	(0.0423)	(0.0459)	(0.471)
ΔGB2Y(t-3)	0.178*	0.0570	-0.0180	0.551
	(0.0945)	(0.0421)	(0.0457)	(0.470)
ΔSELIC(t-1)	0.150	-0.155**	0.0794	-0.0522
	(0.175)	(0.0781)	(0.0848)	(0.871)
ΔSELIC(t-2)	0.316*	0.0651	0.0832	0.295
	(0.173)	(0.0772)	(0.0838)	(0.860)
ΔSELIC(t-3)	0.0546	0.342***	0.0568	-0.604
	(0.171)	(0.0764)	(0.0829)	(0.852)
ΔCPI(t-1)	0.421**	0.0347	0.495***	-0.0401
	(0.190)	(0.0847)	(0.0919)	(0.944)
ΔCPI(t-2)	0.0437	0.00587	-0.0421	-0.124
	(0.209)	(0.0929)	(0.101)	(1.036)
ΔCPI(t-3)	0.237	0.0257	0.142	-0.179
	(0.191)	(0.0853)	(0.0925)	(0.950)
ΔIP(t-1)	0.0277	-0.00352	-0.00861	-0.0345
	(0.0196)	(0.00874)	(0.00949)	(0.0974)
ΔIP(t-2)	0.0579***	-0.00533	0.00863	0.108
	(0.0194)	(0.00864)	(0.00938)	(0.0963)
ΔIP(t-3)	-0.00630	0.00491	0.0117	0.0576
	(0.0194)	(0.00866)	(0.00940)	(0.0965)
Constant	0.0118	-0.0301	0.0132	-0.0108
	(0.0544)	(0.0242)	(0.0263)	(0.270)
Observations	137	137	137	137
P>chi2	0.0198	0.0000	0.0000	0.5280
R-square	0.1807	0.5969	0.3592	0.0962

Notes: Standard errors in parentheses.

*** p<0.01

** p<0.05

* p<0.1.

**Table 8:** Speed of adjustment and short-run relationship from VECM for Eq 7.

VARIABLES	ΔGB10Y	ΔSELIC	ΔCPI	ΔIP
	Speed of Adjustment			
Error Correction	-0.138***	0.0338*	-0.0488***	-0.409**
	(0.0429)	(0.0175)	(0.0176)	(0.181)
	Short-run relationship			
ΔGB10Y(t-1)	0.0509	0.0408	0.0452	0.127
	(0.0885)	(0.0361)	(0.0363)	(0.373)
ΔGB10Y(t-2)	-0.154*	-0.0412	-0.0273	-0.306
	(0.0852)	(0.0348)	(0.0350)	(0.359)
ΔGB10Y(t-3)	0.0928	0.0408	0.0290	0.256
	(0.0881)	(0.0359)	(0.0361)	(0.371)
ΔSELIC(t-1)	0.237	-0.0213	0.143*	0.404
	(0.195)	(0.0796)	(0.0800)	(0.822)
ΔSELIC(t-2)	0.434**	0.149*	0.0847	0.408
	(0.190)	(0.0777)	(0.0781)	(0.803)
ΔSELIC(t-3)	0.0524	0.393***	0.0722	-0.489
	(0.193)	(0.0789)	(0.0794)	(0.815)
ΔCPI(t-1)	0.459**	0.111	0.531***	0.279
	(0.220)	(0.0899)	(0.0904)	(0.929)
ΔCPI(t-2)	-0.0243	0.0748	-0.0109	0.271
	(0.243)	(0.0993)	(0.0999)	(1.026)
ΔCPI(t-3)	0.316	0.0596	0.145	-0.194
	(0.223)	(0.0912)	(0.0917)	(0.942)
ΔIP(t-1)	0.0442*	0.00443	-0.00300	-0.00304
	(0.0228)	(0.00933)	(0.00938)	(0.0963)
ΔIP(t-2)	0.0904***	0.00208	0.0128	0.145
	(0.0224)	(0.00916)	(0.00921)	(0.0946)
ΔIP(t-3)	-0.0225	0.0124	0.0180*	0.115
	(0.0230)	(0.00938)	(0.00943)	(0.0969)
Constant	0.0281	-0.0173	0.0148	-0.0127
	(0.0622)	(0.0254)	(0.0255)	(0.262)
Observations	137	137	137	137
P>chi2	0.0006	0.0000	0.0000	0.3535
R-square	0.2350	0.5391	0.3710	0.1119

Notes: Standard errors in parentheses.

*** p<0.01

** p<0.05

* p<0.1.

Table 7 shows the short-run relationships among variables. Based on the optimal lag length selection, the optimal lag length is three, and there is one cointegration equation. For example, in column 2, there are three lags for each variable. For the variable changes of GB2Y, the third lag of GB2Y is statistically significant, which implies that GB2Y is impacted by its own three prior month’s values. In addition, GB2Y is influenced by SELIC with a two periods lag, CPI with a one-period lag, and IP with a two-period lag. In column 3, most variables are statistically insignificant when the trend term is suppressed in the regression.

Table 8 shows the short-run relationship among variables following Eq 7. Based on the optimal lag length selection, the optimal lag length is three, and there is one cointegration equation. For example, in column 2, for the variable changes of GB10Y, the second lag of GB10Y is statistically significant, which implies that GB10Y is impacted by its own two prior months’ values. SELIC impacts GB10Y yield with a two-period lag, CPI with a one-period lag, and IP with a two-period lag. In column 3, the trend term in the regression is suppressed. Here GB10Y yield is significantly affected by its second lag.

The empirical exercises undertaken with the alternative specification show a positive relationship between government bond yields and the short-run interest rate, as measured by the SELIC rate. These findings hold for both in the front end and the back end of the yield curve. The sign in the short-run to long-run deviation is negative as expected. This finding supports Keynes’s conjecture.

These two different specifications of the models show that generally, there is a positive relationship between the short-term interest rate and the long-term interest rate in Brazil. The findings are consistent with Keynes’s hypothesis that the short-term interest rate has a decisive influence on the long-term interest rate on government bonds. Additional regression results, available in the working paper, obtain similar results and support Keynes’s hypothesis.

## Economic policy implications and conclusion

The empirical results obtained in this paper have economic policy implications for Brazil and other emerging market countries.

The empirical findings clearly show that a higher (lower) short-term swap rate leads to higher (lower) long-term BGB yields, particularly in the front end of the BGB yield curve. The BCB influences BGB yields primarily through the SELIC target rate, affecting the short-term swap rate. The findings models emphasize the role of the swap rate as a key determinant of BGB yields and the shape of the yield curve.

If the SELIC target rate is the primary driver of BGB yields, there are consequential implications for macroeconomics, financial markets, and monetary policy. The findings of the paper suggest BCB’s actions appear to have a decisive effect on BGB yields. The BCB’s policy rate has a marked impact on BGB’s nominal yields through its influence on the swap rate. A higher (lower) short-term interest rate is associated with higher (lower) government bond yields. The BCB influences BGB yields by influencing the policy rate on short-term interest rates, such as the swap rates and the effective SELIC rates. Given its monetary sovereignty, the BCB appears to have the operational ability and flexibility to effectively influence BGBs’ yields on government debt in local currency as necessary, provided that a regime of floating exchange rate is maintained. However, besides the SELIC target rate, the BCB has other monetary policy tools at its disposal, such as calendar-based and information-conditional forward guidance, policy pronouncements, large-scale asset purchases, and yield curve control that can also affect BGB yields.

Besides the influence of the policy rate of BGB yields, the effects of other monetary policy tools should be a future research topic. The influence of the fiscal deficit ratio or the government debt ratio on BGB yields should also be carefully examined, particularly to determine whether the claim of the loanable funds theory has any empirical support. The BCB and other central banks of emerging market countries need to also pay attention to the exchange rate of their currency because the exchange rate because currency depreciation (appreciation) can have marked inflationary (deflationary) effects and real effects on effective demand and economic activity, exports and imports, competitiveness, and the standard of living.

The BCB’s policy rate decision is affected by the statutory mandates, inflationary pressures, inflation expectations, and overall economic and financial conditions and various economic and political constraints. Nevertheless, the findings support the view that the BCB’s monetary policy actions are an important driver of the long-term interest rate and the shape of the yield curve.

The results presented in the paper show that the Keynesian perspective on government bond yields can be useful for modelling the dynamics of the BGB yields. The results generally support that Keynes’s [1: 352–353] contentions that (1) “the long-term rate of interest will respond to the wishes of a Currency Authority which will be exerting its direct influence … mainly on the short-term rate” and (2) “the influence of the short-term rate of interest on the long-term rate is much greater than anyone … would have expected” appear to hold for Brazil.

The findings from the paper can inform policy issues and discussions in Brazil related to BGB yields and the yield curve, government debt management, fiscal sustainability, fiscal policy and the efficacy of monetary policy and monetary transmission mechanism. The findings can also have policy implications for other emerging market countries, particularly in Latin America, which often face economic circumstances and financial markets conditions and institutions similar to those in Brazil. Earlies studies of other emerging markets with currency sovereignty, such as India [30, 32], align with the findings of this paper.

The understanding of the observed behavior patterns of the BGBs yields and its dynamics as provided in this paper can be useful to both long standing debates and ongoing controversies in macroeconomic theory on a wide range of topics, such as the effects of monetary policy, quantitative easing, operational issues in central banking [46, 71], the fiscal theory of price [52, 54], efficient market hypothesis, government debt sustainability [40], fiscal austerity, fiscal policy, fiscal-monetary coordination [53], functional finance [50–51], modern money and chartalism [45], and bond markets in emerging economics [61]. It is hoped that the findings will contribute to promoting sound and welfare-enhancing public policies and further research on key macroeconomic issues in Brazil and other emerging markets. The related literature on government bond market in emerging markets have also examined the influence of various other variables, such as government debt and deficit ratios [62], exchange rate and exchange rate risks [72], market volatility, and other factors, besides the ones considered here. Further research on BGBs can extend the findings of this paper by examining the effects of these and other macroeconomic and financial variables in the context of ongoing developments in Brazil’s economy and the government bond market.

## Supporting information

S1 Dataset (XLSX)

S1 Appendix (DOCX)

S2 Appendix (DOCX)

## References

[1] Keynes JM. A treatise on money, Vol. II: The applied theory of money. London, UK: Macmillan; 1930.

[2] Simoski S. A Keynesian exploration of the determinants of government bond yields for Brazil, Colombia, and Mexico. Master of Science thesis, Levy Economics Institute of Bard College; 2019. https://digitalcommons.bard.edu/levy_ms/16

[3] Ardagna S, Caselli F, Lane T. Fiscal discipline and the cost of public debt service: Some estimates for OECD countries. The B.E. Journal of Macroeconomics. 2007; 7(1): 1–33. 10.2202/1935-1690.1417

[4] Baldacci E, Kumar M. Fiscal deficits, public debt, and sovereign bond yields. IMF Working Paper No. 10/184. 2010. 10.5089/9781455202188.001

[5] Cebula R. An empirical investigation into the impact of US federal government budget deficits on the real interest rate yield on intermediate-term treasury issues, 1972–2012. Applied Economics. 2014; 46(28): 3483–93. 10.1080/00036846.2014.932050

[6] Gruber JW, Kamin SB. Fiscal positions and government bond yields in OECD countries. Journal of Money, Credit, and Banking. 2012; 44(8): 1563–1587. 10.1111/j.1538-4616.2012.00544.x

[7] Horioka CY, Nomoto T, Terada-Hagiwara A. Why has Japan’s massive government debt not wreaked havoc (yet)? The Japanese Political Economy. 2014; 40(2): 3–23. 10.2753/JES2329-194X400201

[8] Hoshi T, Ito T. Is the sky the limit? Can Japanese government bonds continue to defy gravity? Asian Economic Policy Review 2013. 8(2): 218–247. 10.1111/aepr.12023

[9] Hoshi T, Ito T. Defying gravity: Can Japanese sovereign debt continue to increase without a crisis? Economic Policy 2014; 29(77): (January) 5–44. 10.1111/1468-0327.12023

[10] Martinez LB., Tercenoa A, Teruelb M. Sovereign bond spreads determinants in Latin American countries: Before and during the XXI financial crisis. Emerging Markets Review. 2013; 17: 60–75. 10.1016/j.ememar.2013.08.004

[11] Min HG, Lee DH, Nam C, Park MC, Nam SH. Determinants of emerging-market bond spreads: cross-country evidence. Global Finance Journal. 2003; 14(3): 271–286. 10.1016/j.gfj.2003.10.001

[12] Poghosyan T. Long-run and short-run determinants of sovereign bond yields in advanced economies. Economic Systems. 2014; 38(1): 100–114. 10.1016/j.ecosys.2013.07.008

[13] Reinhart CM, Rogoff KS. This time is different: Eight centuries of financial folly. Princeton, NJ: Princeton University Press; 2009.

[14] Tkačevs O, Vilerts K. The impact of sovereign bond yields on fiscal discipline. Latvijas Banka Working Paper No. 5/2016. 2016. https://www.macroeconomics.lv/sites/default/files/2017-02/wp-5_2016-en.pdf

[15] Tkačevs O, Vilerts K. The impact of government borrowing costs on fiscal discipline. Kyklos. 2019; 729(3): 446–471. 10.1111/kykl.12207

[16] Cox JC, Ingersoll JE Jr, Ross SA. A re‐examination of traditional hypotheses about the term structure of interest rates. The Journal of Finance. 1981; 36(4): 769–99. 10.1111/j.1540-6261.1981.tb04884.x

[17] Lutz FA. The structure of interest rates. The Quarterly Journal of Economics. 1940–41; 55(1): 36–63. 10.2307/1881665

[18] Culbertson JM. The term structure of interest rates. The Quarterly Journal of Economics. 1957; 71(4): 485–517. 10.2307/1885708

[19] Hicks JR. Value and capital. Second edition. London, UK: Oxford University Press; 1946.

[20] Campbell JY. A defense of traditional hypotheses about the term structure of interest rates. The Journal of Finance. 1986; 41(1): 183–93. 10.1111/j.1540-6261.1986.tb04498.x

[21] Campbell JY, Shiller RJ. Yield spreads and interest rate movements: A bird’s eye view. The Review of Economic Studies. 1991; 58(3):495–514. 10.2307/2298008

[22] Fama EF. The information in the term structure. Journal of financial economics. 1984; 13(4): 509–28. 10.1016/0304-405X(84)90013-8

[23] Mankiw NG, Summers LH. Do long-term interest rates overreact to short-term interest rates? National Bureau of Economic Research; 1984. Working Paper 1345. http://dx.doi.org.10.3386/w1345

[24] Michaelsen JB. The term structure of interest rates: Comment. The Quarterly Journal of Economics. 1963; 77(1): 166–74. 10.2307/1879382

[25] Fabozzi FJ. The Structure of Interest Rates. In Fabozzi F J (eds). The Handbook of Fixed Income Securities. Fifth edition. New York, NY: McGraw-Hill; 1997. doi: 10.1111/j.1574-6968.1997.tb12624.x

[26] McEnally RW, Jordan JV. The Term Structure of Interest Rates. In Fabozzi F J (eds). The Handbook of Fixed Income Securities. Fifth edition. New York, NY: McGraw-Hill; 1997.

[27] Keynes JM. The general theory of employment, interest, and money. New York, NY: Palgrave Macmillan; 2007 [1936].

[28] Riefler WW. Money rates and money markets in the United States. New York, NY, and London, UK: Harper & Brothers; 1930.

[29] Akram T, Das A. Understanding the low yields of the long-term Japanese sovereign debt. Journal of Economic Issues. 2014; 48(2): 331–340. 10.2753/JEI0021-3624480206

[30] Akram T, Das A. A Keynesian explanation of Indian government bond yields. Journal of Post Keynesian Economics. 2015; 38(4): 565–587. 10.1080/01603477.2015.1090294

[31] Akram T, Das A. The dynamics of government bond yields in the eurozone. Annals of Financial Economics. 2017; 12(3): 1750011–18. 10.1142/S2010495217500117

[32] Akram T, Das A. The long-run determinants of Indian government bond yields. Asian Development Review. 2019a; 36(1): 168–205. 10.1162/adev_a_00127

[33] Akram T, Das A. An analysis of the daily changes in U.S. treasury security yields. Levy Economics Institute Working Paper No. 934. 2019b. http://www.levyinstitute.org/pubs/wp_934.pdf

[34] Akram T, Das A. Australian government bonds’ nominal yields: A Keynesian perspective. Annals of Financial Economics. 2020; 15(1): 2050003-1-2050003-20. 10.1142/S2010495220500037

[35] Akram T, Li H. The empirics of long-term U.S. interest rates. Levy Economics Institute Working Paper No. 863. 2016. http://www.levyinstitute.org/pubs/wp_863.pdf

[36] Akram T, Li H. What keeps long-term U.S. interest rates so low? Economic Modelling. 2017; 60: 380–390. 10.1016/j.econmod.2016.09.017

[37] Akram T, Li H. The impact of the Bank of Japan’s monetary policy on Japanese Government Bonds’ Low Nominal Yields. Levy Economics Institute Working Paper No. 938. 2019. http://www.levyinstitute.org/pubs/wp_938.pdf

[38] Akram T, Li H. An inquiry concerning long-term U.S. interest rates using monthly data. Applied Economics. 2020a; 52(24): 2594–2621. 10.1080/00036846.2019.1693696

[39] Akram T, Li H. JGBs’ chronically low nominal yields: A VEC approach. Applied Economics. 2020b; 52(53): 5873–5893. 10.1080/00036846.2020.1776838

[40] Fullwiler ST. The debt ratio and sustainable macroeconomic policy. World Economic Review. 2016; 7: 12–42. http://wer.worldeconomicsassociation.org/files/WEA-WER-7-Fullwiler.pdf

[41] Kregel J.Was Keynes’ monetary policy à outrance in the Treatise, a forerunner of ZIRP and QE?Did he change his mind in the General Theory? Levy Economics Institute, Policy Note No. 2011/4. 2011. http://www.levyinstitute.org/pubs/pn_11_04.pdf

[42] Lavoie M. Post-Keynesian economics: New foundations. Cheltenham, UK, and Northampton, MA: Edward Elgar; 2014.

[43] Deleidi M, Levrero ES. Monetary policy and long‐term interest rates: Evidence from the US economy. Metroeconomica. 2021; 72(1): 121–147. 10.1111/meca.12313

[44] Wray LR. Understanding modern money: The key to full employment and price stability. Paperback edition. Cheltenham, UK, and Northampton, MA: Edward Elgar; 2003 [1998].

[45] Wray LR. Modern money theory: A primer on macroeconomics for sovereign monetary systems. New York, NY: Palgrave Macmillan; 2012.

[46] Bindseil U. Monetary policy implementation: Theory, past, and present. Oxford, UK, and New York, NY: Oxford University Press; 2004. doi: 10.1016/j.pt.2004.05.009

[47] Davidson P. Post Keynesian theory and policy: A realistic analysis of the market oriented capitalist economy. Cheltenham, UK, and Northampton, MA: Edward Elgar; 2015.

[48] Goodhart CAE. Two concepts of money: Implications for the analysis of optimal currency areas. European Journal of Political Economy. 1998; 14(3): 407–432. 10.1016/S0176-2680(98)00015-9

[49] Knapp GF. The state theory of money. Clifton, NY: A.M. Kelley; 1973 [1926].

[50] Lerner AP. Functional finance and the federal debt. Social Research. 1943; 10(1): 38–51. https://www.jstor.org/stable/40981939

[51] Lerner AP. Money as a creature of the state. American Economic Review. 1947; 37(2): 312–17. https://www.jstor.org/stable/1821139

[52] Sims CA. Paper money. American Economic Review. 2013; 103(2): 563–584. http://dx.doi.org/10/1257/aer.103.2.563

[53] Tcherneva PR. Bernanke’s paradox: Can he reconcile his position on the federal budget with his recent charge to prevent deflation? Journal of Post Keynesian Economics. 2011; 33(3): 411–434. 10.2753/PKE0160-3477330301

[54] Bölükbaş M.The fiscal theory of the price level: An analysis for fragile countries. In İsmail Şiriner and Zişan Yardım Kiliçkan, eds., Institutions, Development & Economic Growth. London, UK: IJOPEC; 2018.

[55] Kurihara Y. Asset price and monetary policy: The Japanese case. Journal of Applied Finance and Banking. 2015; 5(4):1–9. https://www.scienpress.com/journal_focus.asp?main_id=56&Sub_id=IV&Issue=1531

[56] Malliaropulos D, Migiakis P. Quantitative easing and sovereign bond yields: A global perspective. Bank of Greece Working Paper, No. 253. 2018. https://www.bankofgreece.gr/BogEkdoseis/Paper2018253.pdf

[57] Mattos OB, Da Roz F, Ultremare F O, Mello G S. Unconventional monetary policy and negative interest rates: A Post-Keynesian perspective on the liquidity trap and euthanasia of the rentier. Review of Keynesian Economics. 2019; 7(2): 185–200. 10.4337/roke.2019.02.05

[58] Patra MD, Pattanaik S, John J, Behera HK. Monetary policy transmission in India: Do global spillovers matter? RBI Occasional Papers. 2016; 37(1&2). https://m.rbi.org.in/Scripts/bs_viewcontent.aspx?Id=3477

[59] Sau L. Coping with deflation and the liquidity trap in the Eurozone: A Post Keynesian approach. Journal of Post Keynesian Economics. 2018; 41(2): 210–235. 10.1080/01603477.2017.1387498

[60] Jaramillo L, Weber A. Bond yields in emerging economies: It matters what state you are in. Emerging Markets Review 2013; 17: 169–185. 10.1016/j.ememar.2013.09.003

[61] Turner, P. Bond markets in emerging economies: An overview of policy issues. BIS Papers No. 11. 2002. https://www.bis.org/publ/bppdf/bispap11b.pdf

[62] Eichengreen B, Mody A. What explains changing spreads on emerging-market debt: Fundamentals or market sentiment? National Bureau of Economic Research Working Paper No. 6408. 1998. https://www.nber.org/system/files/working_papers/w6408/w6408.pdf

[63] Cardim de Carvalho FJ. Looking into the abyss? Brazil at the mid-2010s. Journal of Post Keynesian Economics. 2016a; 39(1): 93–114. 10.1080/01603477.2016.1152556

[64] Cardim de Carvalho FJ. The narrow path for Brazil. Policy Note 2016/2. 2016b. http://www.levyinstitute.org/pubs/pn_16_2.pdf

[65] Cardim de Carvalho FJ. Brazil still in troubled waters. Public Policy Brief No. 2017/143. 2017. http://www.levyinstitute.org/pubs/ppb_143.pdf

[66] International Monetary Fund. Article IV consultation—press release; staff report; and statement by the Executive Director for Brazil. IMF Country Report No. 19/242. 2019a. https://www.imf.org/-/media/Files/Publications/CR/2019/1BRAEA2019001.ashx

[67] International Monetary Fund. Brazil: Staff report for the 2019 Article IV consultation. IMF Country Report No. 19/243. 2019b. https://www.imf.org/en/Publications/CR/Issues/2019/07/23/Brazil-Selected-Issues-48521

[68] Kregel J. The global crisis and the implications for developing countries and the BRICS: Is the ‘B’ really justified? Brazilian Journal of Political Economy. 2009; 29(4): 341–356. http://www.scielo.br/pdf/rep/v29n4/02.pdf

[69] Macrobond. Macrobond subscription services. Various years. https://www.macrobond.com (accessed November 10, 2019).

[70] Johansen S. Likelihood-based inference in cointegrated vector autoregressive models. Oxford, UK: Oxford University Press; 1995. 10.1093/0198774508.001.0001

[71] Fullwiler ST. Modern central bank operations: The general principles. In Louis-Philippe Rochon and Sergio Rossi, ed., Advances in endogenous money analysis. Northampton, MA: Edward Elgar; 2017 [2008].

[72] Schmukler SL, Servén L. Pricing currency risk under currency boards. Journal of Development Economics. 2002; 69(2): 367–391. 10.1016/S0304-3878(02)00093-7

