# Supplementary material for: An empirical analysis of long-term Brazilian interest rates
Source: PLoS One. 2021 Sep 10;16(9):e0257313. doi: 10.1371/journal.pone.0257313 (PMC8432776; doi:10.1371/journal.pone.0257313)
Supplement: S1 Appendix — (DOCX) [file pone.0257313.s002.docx]

**S1 Appendix**

**Unit roots tests**

Unit root and stationary properties of each of the variables are checked. The augmented Dickey-Fuller (ADF) [1-2] and Philips-Perron (PP) [3] tests are to determine the unit root properties of the data. The lag length of the tests is based on the Akaike information criterion (AIC). The generalized ADF test also confirms the lag length choice.

Table A1 presents the results of ADF tests. It contains the three types of ADF tests: (1) random walk with drift ($\delta=0$), (2) random walk with or without drift (none), and (3) random walk without drift ($\alpha=0, \delta=0$). In the ADF test, the null hypothesis is always that the variable has a unit root. Table A1 shows that the null hypothesis of unit root cannot be rejected for most variables at 1 percent and 5 percent levels of significance. However, the null hypothesis of unit root for the first difference of most variables can be rejected at 1 percent and 5 percent levels of significance.

**Table A1: Augmented Dickey-Fuller (ADF) unit root testing results.**

|  | ADF test in level | | ADF test in first difference | | | |  | |  |
| --- | --- | --- | --- | --- | --- | --- | --- | --- | --- |
| Variables | Test Types | DF stat | | Variables | | DF Stat | | Integration Order | |
| GB2Y | Drift | -1.520 | ΔGB2Y | | -4.985*** | | I(1) | |  |
| GB2Y | Trend | -1.920 | ΔGB2Y | | -5.029*** | | I(1) | |  |
| GB2Y | No-trend and Drift | -0.669 | ΔGB2Y | | -4.988*** | | I(1) | |  |
| SWAP30D | Drift | -2.315* | ΔSWAP30D | | -3.702*** | | I(1) | |  |
| SWAP30D | Trend | -2.414 | ΔSWAP30D | | -3.723*** | | I(1) | |  |
| SWAP30D | No-trend and Drift | -0.838 | ΔSWAP30D | | -3.694*** | | I(1) | |  |
| GPI | Drift | -2.218* | ΔGPI | | -3.846*** | | I(1) | |  |
| GPI | Trend | -2.437 | ΔGPI | | -3.827*** | | I(1) | |  |
| GPI | No-trend and Drift | -0.938 | ΔGPI | | -3.870*** | | I(1) | |  |
| GDP | Drift | -2.207 | ΔGDP | | -5.819*** | | I(1) | |  |
| GDP | Trend | -2.325 | ΔGDP | | -5.799*** | | I(1) | |  |
| GDP | No-trend and Drift | -2.194 | ΔGDP | | -5.835*** | | I(1) | |  |
| GB5Y | Drift | -1.403 | ΔGB5Y | | -3.520*** | | I(1) | |  |
| GB5Y | Trend | -1.378 | ΔGB5Y | | -3.567*** | | I(1) | |  |
| GB5Y | No-trend and Drift | -0.613 | ΔGB5Y | | -3.526*** | | I(1) | |  |
| GB10Y | Drift | -2.306 | ΔGB10Y | | -9.535*** | | I(1) | |  |
| GB10Y | Trend | -2.649 | ΔGB10Y | | -9.568*** | | I(1) | |  |
| GB10Y | No-trend and Drift | -0.602 | ΔGB10Y | | -9.569*** | | I(1) | |  |

Notes: ADF stat presents the calculated statistic for the variables following the Augmented Dickey-Fuller model with the optimal lag length. Lag length is selected based on AIC. Here drift means that the process under the null hypothesis is a random walk with drift (unit root with drift), so the population value of $\alpha$ is non-zero. Trend means the process under the null hypothesis is a unit root with or without drift so that $\alpha$ is unrestricted and includes a time trend in the regression. No trend, no drift means that the process under the null hypothesis is a unit root without drift. It fits without any constant term $\alpha$ and any time trend $\delta t$.

**Table A2. Phillips-Perron (PP) unit roots test.**

|  | PP test in level | | PP Test in first difference | |  |
| --- | --- | --- | --- | --- | --- |
| Variables | Test Type | Test Stat | Variables | Test Stat | Integration Order |
| GB2Y | No Constant | -0.719 | ΔGB2Y | -10.42*** | I(1) |
| GB2Y | Trend | -1.998 | ΔGB2Y | -10.42*** | I(1) |
| SWAP30D | No Constant | -1.100 | ΔSWAP30D | -4.139*** | I(1) |
| SWAP30D | Trend | -1.407 | ΔSWAP30D | -4.169*** | I(1) |
| GPI | No Constant | -1.262* | ΔGPI | -5.114*** | I(1) |
| GPI | Trend | -2.707 | ΔGPI | -5.070*** | I(1) |
| GDP | No Constant | -2.235* | ΔGDP | -10.09*** | I(1) |
| GDP | Trend | -2.469 | ΔGDP | -10.05*** | I(1) |
| GB5Y | No Constant | -0.707 | ΔGB5Y | -8.243*** | I(1) |
| GB5Y | Trend | -1.499 | ΔGB5Y | -8.217*** | I(1) |
| GB10Y | No Constant | -0.493 | ΔGB10Y | -11.75*** | I(1) |
| GB10Y | Trend | -2.948 | ΔGB10Y | -11.73*** | I(1) |

Note: PP statistics presents the calculated statistic for the variables following the Phillips-Perron (PP) model with the optimal lag length. Lag length is selected based on the AIC.

Table A2 presents the result of the Phillips-Perron (PP) tests. It contains two types of PP tests: (1) random walk without drift ($\alpha=0, \delta=0$) and (2) random walk with or without drift (none). The null hypothesis is that the variable contains a unit root, while the alternative hypothesis is that the variable was generated by a stationary process. Table A2 shows the null hypothesis of unit root cannot rejected for most variables at 1 percent and 5 percent levels of confidence. However, the null hypothesis of unit root for the first difference of most variable can be rejected at 1 percent and 5 percent levels of confidence.

**Lag-length and cointegration tests**

The cointegration test is applied to determine whether there is really a long-run (or cointegrating) relationship among the variables. The optimal lag length is selected for the cointegration tests. Table A3 reports the log-likelihood (LL), likelihood ratio (LR), final prediction error (FPE), Akaike’s information criterion (AIC), Schwarz’s Bayesian information criterion (SBIC), and the Hannan and Quinn information criterion (HQIC) lag-order selection statistics for a series of vector autoregressions of order 1 through a requested maximum lag. For a given lag *p*, the LR test compares a VAR with *p* lags with one with *p-1* lags. The null hypothesis is that all the coefficients on the *p*th lags of the endogenous variables are zero. To use this sequence of LR tests to select a lag order, the results of the test for the model with the most lags, which is at the bottom of the table, were carefully examined. This paper uses the AIC as the selection criteria for finding the optimal lag length. However, the results of the paper do not materially change if instead other information criteria are used for determining the optimal lag length.

**Table A3. Lag length selection.**

| lag | **LL** | **LR** | **FPE** | **AIC** | **HQIC** | **SBIC** |
| --- | --- | --- | --- | --- | --- | --- |
| Part A: Variables: GB2Y, SWAP30D GPI, GDP | | | | | | |
| 0 | -1264.847 |  | 1302.189 | 18.523 | 18.558 | 18.609 |
| 1 | -498.880 | 1531.934 | 0.023 | 7.575 | 7.748 | 8.001 |
| 2 | -428.421 | 140.918 | 0.010 | 6.780 | 7.092* | 7.547* |
| 3 | -409.863 | 37.117 | 0.010* | 6.743* | 7.193 | 7.851 |
| 4 | -394.391 | 30.944* | 0.010 | 6.750 | 7.339 | 8.200 |
| Part B: Variables: GB2Y, SWAP30D, GPI | | | | | | |
| 0 | -898.855 |  | 104.808 | 13.166 | 13.192 | 13.230 |
| 1 | -300.570 | 1196.570 | 0.019 | 4.563 | 4.667 | 4.819 |
| 2 | -230.451 | 140.239 | 0.008 | 3.671 | 3.853* | 4.118* |
| 3 | -218.957 | 22.987 | 0.008 | 3.634 | 3.894 | 4.274 |
| 4 | -209.224 | 19.466* | 0.008* | 3.624* | 3.962 | 4.455 |
| Part C: Variables: GB10Y, SWAP30D, GPI, GDP | | | | | | |
| 0 | -1270.875 |  | 1421.983 | 18.611 | 18.646 | 18.697 |
| 1 | -555.380 | 1430.990 | 0.052 | 8.400 | 8.573 | 8.826 |
| 2 | -464.139 | 182.481 | 0.017 | 7.301 | 7.613* | 8.069* |
| 3 | -440.188 | 47.903 | 0.016* | 7.185* | 7.636 | 8.294 |
| 4 | -424.824 | 30.728* | 0.016 | 7.195 | 7.783 | 8.644 |
| Part D: Variables: GB10Y, SWAP30D, GPI | | | | | | |
| 0 | -897.485 |  | 102.734 | 13.146 | 13.172 | 13.210 |
| 1 | -362.876 | 1069.218 | 0.048 | 5.473 | 5.577 | 5.728 |
| 2 | -266.366 | 193.020 | 0.013 | 4.195 | 4.377 | 4.643* |
| 3 | -251.123 | 30.486* | 0.012* | 4.104* | 4.364* | 4.743 |
| 4 | -242.926 | 16.395 | 0.012 | 4.116 | 4.454 | 4.947 |

Note: An ‘*’ appears next to the LR and other statistic indicating the optimal lag. The lag with the smallest value is the order selected by the information criterion.

Table A3 presents various measures of lag length selection criteria. It is evident from the table A3 (part A) that based on the AIC, the optimal lag length is 3. However, the Hannan-Quinn information criterion (HQIC) and the Schwarz Bayesian information criterion (SIBC) method suggested 2 lags. Similarly, in part B, GDP was dropped from the VAR list. The remaining three variables with an optimal lag length of 4, based on AIC, were used. In part C, GB2Y was replaced with GB10Y keeping other variables same as parts A and B. Following the AIC, 3 lags were used in both in parts C and D.

Following the optimal lag length, the cointegration relationship is defined based on Johansen [4]. If all variables in $Y_{t}$ are I(1), the matrix Π has rank 0 ≤ *r* < *K*, where *r* is the number of linearly independent cointegrating vectors. If the variables are cointegrated (*r* > 0) the VAR in first differences is mis-specified, as it excludes the error correction term.

The table A4 reports the rank test for cointegrating equation. Table A4, part A, shows the Johansen test statistics for rank test. The first is Johansen’s “trace” statistic method. The second is the “maximum eigenvalue” statistic method. According to the trace statistic test, for any given value of r, large values of the trace statistic are evidence against the null hypothesis that there is r or fewer cointegrating relations in the VECM. In the table A4, each row represents one hypothesis test. For each test it reports the maximum rank under the null, the number of parameters estimated, the log-likelihood, the r-th eigenvalue, the trace statistic, and a 5% critical value for the trace statistic. Trace statistic confirms that here r =1, which implies there is only one cointegrating equation. In this example, the trace statistic at r = 0 of 69.417 exceeds its critical value of 62.990. Hence, the null hypothesis of no cointegrating equations is rejected. Similarly, the trace statistic at r = 1 of 35.091 is less than its critical value of 42.440. Hence, the null hypothesis that there are one or fewer cointegrating equations cannot be rejected. The “*” by the trace statistic at r = 1 indicates that this is the value of r selected by Johansen’s multiple-trace test procedure. The eigenvalue shown in the last line of output computes the trace statistic in the preceding line. When max statistic is lower than the 5% critical value, the null hypothesis is rejected.

In table A4 (part B) the variable GDP is dropped. The trace statistic shows that r = 1, which implies that there is one cointegrating equation. Similarly, in part C and D, for GB10Y trace statistic suggests that there is one cointegrating relationship for each model.

**Table A4. Rank of the cointegration order (Johansen test [4]).**

| Part A: Variables: GB2Y, SWAP30D, GPI, GDP | | | | | |
| --- | --- | --- | --- | --- | --- |
| Maximum rank | Parms | LL | Eigenvalue | Trace statistic | 5% critical value |
| 0 | 36 | -436.017 |  | 69.417 | 62.990 |
| 1 | 44 | -418.856 | 0.220 | 35.095* | 42.440 |
| 2 | 50 | -410.124 | 0.119 | 17.631 | 25.320 |
| 3 | 54 | -403.415 | 0.093 | 4.213 | 12.250 |
| 4 | 56 | -401.308 | 0.030 |  |  |
| Maximum rank | Parms | LL | Eigenvalue | Max statistic | 5% critical value |
| 0 | 36 | -436.017 |  | 34.322 | 31.460 |
| 1 | 44 | -418.856 | 0.220 | 17.464 | 25.540 |
| 2 | 50 | -410.124 | 0.119 | 13.418 | 18.960 |
| 3 | 54 | -403.415 | 0.093 | 4.213 | 12.520 |
| 4 | 56 | -401.308 | 0.030 |  |  |
| Part B: Variables: GB2Y, SWAP30D, GPI | | | | | |
| Maximum rank | Parms | LL | Eigenvalue | Trace statistic | 5% critical value |
| 0 | 21 | -239.211 |  | 48.807 | 42.440 |
| 1 | 27 | -224.961 | 0.187 | 20.3069* | 25.320 |
| 2 | 31 | -216.926 | 0.110 | 4.237 | 12.250 |
| 3 | 33 | -214.807 | 0.030 |  |  |
| Maximum rank | Parms | LL | Eigenvalue | Max statistic | 5% critical value |
| 0 | 21 | -239.211 |  | 28.500 | 25.540 |
| 1 | 27 | -224.961 | 0.187 | 16.070 | 18.960 |
| 2 | 31 | -216.926 | 0.110 | 4.237 | 12.520 |
| 3 | 33 | -214.807 | 0.030 |  |  |
| Part C: GB10Y, SWAP30D, GPI, GDP | | | | | |
| Maximum rank | Parms | LL | Eigenvalue | Trace statistic | 5% critical value |
| 0 | 36 | -466.647 |  | 64.868 | 62.990 |
| 1 | 44 | -451.625 | 0.196 | 34.824* | 42.440 |
| 2 | 50 | -442.863 | 0.119 | 17.300 | 25.320 |
| 3 | 54 | -436.494 | 0.088 | 4.561 | 12.250 |
| 4 | 56 | -434.214 | 0.033 |  |  |
| Maximum rank | Parms | LL | Eigenvalue | Max statistic | 5% critical value |
| 0 | 36 | -466.647 |  | 30.044 | 31.460 |
| 1 | 44 | -451.625 | 0.196 | 17.524 | 25.540 |
| 2 | 50 | -442.863 | 0.119 | 12.739 | 18.960 |
| 3 | 54 | -436.494 | 0.088 | 4.561 | 12.520 |
| 4 | 56 | -434.214 | 0.033 |  |  |
| Part D: GB10Y, SWAP30D, GPI | | | | | |
| Maximum rank | Parms | LL | Eigenvalue | Trace statistic | 5% critical value |
| 0 | 21 | -271.953 |  | 46.222 | 42.440 |
| 1 | 27 | -258.807 | 0.173 | 19.929* | 25.320 |
| 2 | 31 | -251.236 | 0.104 | 4.787 | 12.250 |
| 3 | 33 | -248.843 | 0.034 |  |  |
| Maximum rank | Parms | LL | Eigenvalue | Max statistic | 5% critical value |
| 0 | 21 | -271.953 |  | 26.293 | 25.540 |
| 1 | 27 | -258.807 | 0.173 | 15.141 | 18.960 |
| 2 | 31 | -251.236 | 0.104 | 4.787 | 12.520 |
| 3 | 33 | -248.843 | 0.034 |  |  |

**References**

1. Dickey DA, Fuller WA. Distribution of the estimators for autoregressive time series with a unit root. Journal of the American Statistical Association. 1979; 74(366): 427-431. http://dx.doi.org/10.1080/01621459.1979.10482531
2. Dickey DA, Fuller WA. Likelihood ratio statistics for autoregressive time series with a unit root. Econometrica. 1981; 49(4): 1057-1072. <http://www.jstor.org/stable/1912517>
3. Phillips PCB, Perron P. Testing for a unit root in time series regression. Biometrika. 1988; 75(2): 335-346. http://dx.doi.org/10.1093/biomet/75.2.335
4. Johansen S. Likelihood-based inference in cointegrated vector autoregressive models. Oxford, UK: Oxford University Press; 1995. https://doi.org/10.1093/0198774508.001.0001
