# Supplementary material for: An empirical analysis of long-term Brazilian interest rates
Source: PLoS One. 2021 Sep 10;16(9):e0257313. doi: 10.1371/journal.pone.0257313 (PMC8432776; doi:10.1371/journal.pone.0257313)
Supplement: S2 Appendix — (DOCX) [file pone.0257313.s003.docx]

**S2 Appendix**

This appendix provides the results of various post estimation statistical checks. This includes the structural break tests for the equations; autocorrelation tests for the residuals; multicollinearity tests among the variables; normality test for the residuals; stability tests; and bidirectional Granger causality tests.

**Autocorrelation and Multicollinearity**

Lagrange multiplier tests for autocorrelation are provided here. This test performs the Johansen based Lagrange multiplier (LM) test, as articulated in [1], for autocorrelation in the residuals of vector error-correction models (VECMs). The test is performed at lags j = 1, . . . , j. For each j, the null hypothesis of the test is that there is no autocorrelation at lag j. Based on the lag length selection provided in appendix A, the maximum lag has been specified as 3. At third lag, at the 5% level, the null hypothesis that there is no autocorrelation in the residuals for any of the orders tested cannot be rejected. Thus, this test finds no evidence of model misspecification (Table B.1).

**Table B.1. Test for residuals autocorrelation.**

|  | Model 1 | | Model 2 | |
| --- | --- | --- | --- | --- |
| Lags | Chi2 | Prob > chi2 | Chi2 | Prob > chi2 |
| 1 | 25.68 | 0.0587 | 8.60 | 0.4747 |
| 2 | 21.40 | 0.1635 | 17.62 | 0.0398 |
| 3 | 18.78 | 0.2802 | 10.16 | 0.3377 |

Note: H_0_: no autocorrelation

Next, the correlations among the variables are checked to find the association among the variables. There is no substantial evidence of multicollinearity problems among the variables. Furthermore, the mean of variance inflation factor is 1.32 (table B.2), and the VIF for none of the individual variables is more than 5. This suggests that the multicollinearity does not pose a problem for the models considered here.

**Table B.2. Multicollinearity test (Variance inflation factor).**

| **Variables** | **VIF** | **1/VIF** |
| --- | --- | --- |
| SWAP30D | 1.48 | 0.674162 |
| GPI | 1.32 | 0.759110 |
| GDP | 1.15 | 0.872024 |
| Mean VIF | 1.32 |  |

**Normality**

Jarque–Bera (JB) statistics is computed to test the null hypothesis that the residuals are normally distributed. The JB results present test statistics for each equation and for all equations jointly against the null hypothesis of normality. For the individual equations, the null hypothesis is that the disturbance term in that equation has a univariate normal distribution. For all equations jointly, the null hypothesis is that the K disturbances come from a K-dimensional normal distribution. In the table B.3, the single-equation and overall Jarque–Bera statistics reject the null of normality. However, the non-normality of residuals in time series models of financial data are not unusual.

**Table B.3. Normality Test: Jarque-Bera test.**

|  | Equation 3 | | Equation 4 | |
| --- | --- | --- | --- | --- |
|  | Chi2 | Prob > chi2 | Chi2 | Prob > chi2 |
| ΔGB10Y | 191.036 | 0.0000 | 185.935 | 0.4747 |
| ΔSWAP30D | 8.501 | 0.0142 | 4.338 | 0.0398 |
| ΔGPI | 1.114 | 0.5728 | 0.368 | 0.3377 |
| ΔGDP | 2.417 | 0.2987 |  |  |
| ALL | 203.068 | 0.0000 | 190.641 | 0.0000 |

**Stability**

The stability test for the parameters is undertaken. This test uses the coefficient estimates from the previously fitted VECM to back out estimates of the coefficients of the corresponding VAR and then compute the eigenvalues of the companion matrix. The cointegrating equations be stationary and that the number of cointegrating equations be correctly specified. Although the methods implemented in the rank test identify the number of stationary cointegrating equations, they assume that the individual variables are I(1). This test indicates whether the number of cointegrating equations is mis-specified or whether the cointegrating equations, which are assumed to be stationary, are not stationary. Fig B1 and B2 show only one root is on the circle and no other roots are very close to the surface of the circle. Therefore, it be concluded that there is no stationarity problem in the cointegrating series.

**Fig B1. Stability condition check of VECM estimates for equation 1.**

**Fig B2. Stability condition check of VECM estimates for equation 3.**

**Structural breaks**

The detection of structural changes for a time series object, particularly in linear regression relationship, has been an important topic in econometric research. The most important classes of tests on structural change are the tests from the generalized fluctuation test framework [2] and tests based on F statistics [3-6]. Recently [7-9] have developed multiple structural break tests for a cointegrated regression augmenting several pioneering work [10-11]. Here, both the OLS-CUSUM and recursive CUSUM tests are undertaken to check if there any indication of structural breaks in the models. The results, displayed in Table B4, suggests that models considered here are not impaired by structural breaks. Figures B3-B4 show the evolution of the recursive CUSUM statistic. The cumulative sum of the residuals is applied to identify a potential structural break. These results suggest that the data do not contain a structural break.

**Table B.4. CUSUM test scores.**

|  | Equation 1 | | Equation 3 | |
| --- | --- | --- | --- | --- |
|  | Chi2 | Prob > chi2 | Chi2 | Prob > chi2 |
| Rec CUSUM | 1.142 | 0.0101 | 1.142 | 0.0101 |
| OLS CUSUM | 1.680 | 0.0071 | 1.680 | 0.0071 |

**Fig B3. Recursive CUSUM test for residuals based on equation 1.**


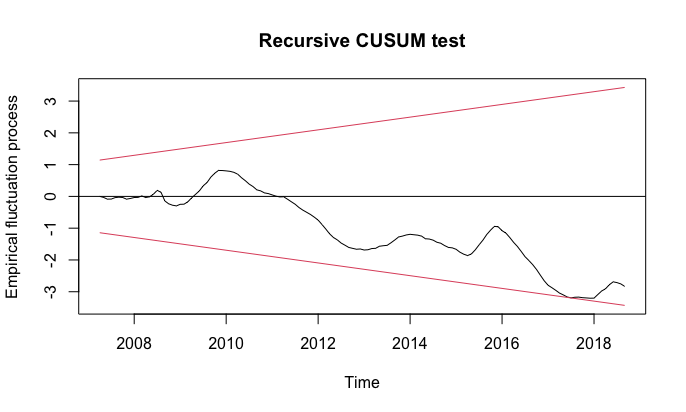


**Fig B4.** **Recursive CUSUM test for residuals based on equation 3.**


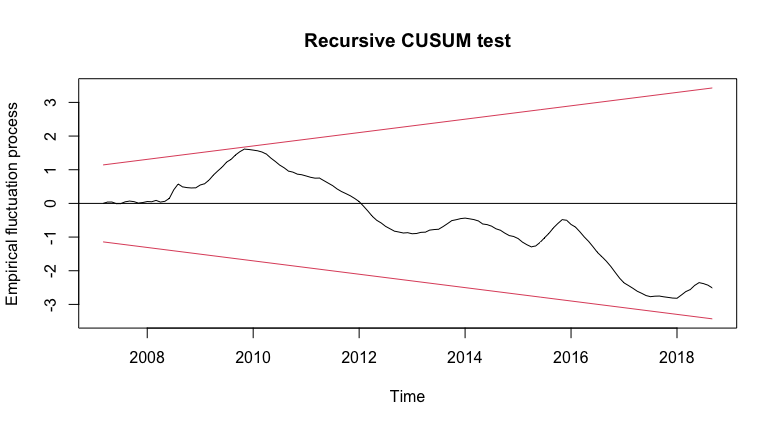


**Bidirectional Granger causality tests**

The bidirectional Granger causality tests are conducted for the VECM model. Table B.5 presents the results from these tests. According to [12], causality can be further sub-divided into long-run and short-run temporal causality. The error correction term determines long-run Granger temporal causality. If it is significant, it indicates evidence of long-run Granger causality from the explanatory variable to the dependent variable. Short-run Granger causality is determined with a test on the joint significance of the lagged explanatory variables, using an F-test or Wald test. The table shows that per Granger tests, SWAP30D “causes” GB10Y, GPI, and GB10Y “causes” SWAP30D and GDP, while GDP does not “cause” SWAP30D, GB10Y, and GPI in the short-run

**Table B.4. Short-run bi-directional Granger causality.**

| **Direction of Causality** | **Decision** |
| --- | --- |
| Does GB10Y cause SWAP30D? | Yes |
| Does GB10Y cause GPI? | No |
| Does GB10Y cause GDP? | Yes |
| Does SWAP30D cause GB10Y? | Yes |
| Does SWAP30D cause GPI? | Yes |
| Does SWAP30D cause GDP? | Yes |
| Does GPI cause GB10Y? | Yes |
| Does GPI cause SWAP30D? | Yes |
| Does GPI cause GDP? | No |
| Does GDP cause SWAP30D? | No |
| Does GDP cause GB10Y? | No |
| Does GDP cause GPI? | No |

**References**

1. Johansen S. Likelihood-based inference in cointegrated vector autoregressive models. Oxford, UK: Oxford University Press; 1995. https://doi.org/10.1093/0198774508.001.0001
2. Kuan CM, Hornik K The generalized fluctuation test: A unifying view. Econometric Reviews. 1995; 14(2): 135-161. https://doi.org/10.1080/07474939508800311
3. Hansen BE. Testing for parameter instability in linear models. Journal of policy modeling. 1992; 14(4): 517-533. <https://doi.org/10.1016/0161-8938(92)90019-9>.
4. Andrews DW. Tests for parameter instability and structural change with unknown change point. Econometrica: Journal of the Econometric Society. 1993; 61(4): 821-856. <https://doi.org/10.2307/2951764>
5. Andrews DW, Ploberger W. Optimal tests when a nuisance parameter is present only under the alternative. Econometrica: Journal of the Econometric Society. 1994; 62(6): 1383-1414. <https://doi.org/10.2307/2951753>
6. Quintos CE. Stability tests in error correction models. Journal of Econometrics. 1998; 82(2): 289-315. https://doi.org/10.1016/S0304-4076(97)00059-6
7. Bergamelli M, Bianchi A, Khalaf L, Urga G. Combining p-values to test for multiple structural breaks in cointegrated regressions. Journal of Econometrics. 2019; 211(2): 461-482. https://doi.org/10.1016/j.jeconom.2019.01.013
8. Bergamelli M, Urga G. Detecting multiple structural breaks: Dummy saturation vs sequential bootstrapping with an application to the Fisher relationship for US. Cass Business School, Working Paper Series No. WP–CEA–03–2014. 2014.
9. Preuss P, Puchstein R, Dette H. Detection of multiple structural breaks in multivariate time series. Journal of the American Statistical Association. 2015; 110(510): 654-68. <https://doi.org/10.1080/01621459.2014.920613>
10. Hansen PR. Structural changes in the cointegrated vector autoregressive model. Journal of Econometrics. 2003; 114(2): 261-295. <https://doi.org/10.1016/S0304-4076(03)00085-X>
11. Bai J, Perron P. Computation and analysis of multiple structural change models. Journal of applied econometrics. 2003; 18(1): 1-22. <https://doi.org/10.1002/jae.659>
12. Granger C. Investigating causal relations by econometric models and cross-spectral methods. Econometrica: Journal of the Econometric Society. 1969; 37(3): 424-438. https://doi.org/10.2307/1912791
